# Ethnic and Racial Variation in Intracerebral Hemorrhage Risk Factors and Risk Factor Burden

**DOI:** 10.1001/jamanetworkopen.2021.21921

**Published:** 2021-08-23

**Authors:** Steven J. Kittner, Padmini Sekar, Mary E. Comeau, Christopher D. Anderson, Gunjan Y. Parikh, Tachira Tavarez, Matthew L. Flaherty, Fernando D. Testai, Michael R. Frankel, Michael L. James, Gene Sung, Mitchell S. V. Elkind, Bradford B. Worrall, Chelsea S. Kidwell, Nicole R. Gonzales, Sebastian Koch, Christiana E. Hall, Lee Birnbaum, Douglas Mayson, Bruce Coull, Marc D. Malkoff, Kevin N. Sheth, Jacob L. McCauley, Jennifer Osborne, Misty Morgan, Lee A. Gilkerson, Tyler P. Behymer, Stacie L. Demel, Charles J. Moomaw, Jonathan Rosand, Carl D. Langefeld, Daniel Woo

**Affiliations:** 1 Geriatric Research and Education Clinical Center, Department of Neurology, Baltimore Veterans Administration Medical Center, University of Maryland School of Medicine, Baltimore; 2 Department of Neurology and Rehabilitation Medicine, University of Cincinnati College of Medicine, Cincinnati, Ohio; 3 Department of Biostatistics and Data Science, Wake Forest University, Winston-Salem, North Carolina; 4 Henry and Allison McCance Center for Brain Health and Center for Genomic Medicine, Massachusetts General Hospital, Boston; 5 Department of Neurology, University of Maryland School of Medicine, Baltimore; 6 Department of Neurological Sciences, Rush University Medical Center, Chicago, Illinois; 7 Department of Neurology and Rehabilitation Medicine, University of Illinois College of Medicine, Chicago, Illinois; 8 Department of Neurology, Emory University, Grady Memorial Hospital, Atlanta, Georgia; 9 Departments of Anesthesiology and Neurology, Duke University, Durham, North Carolina; 10 Neurocritical Care and Stroke Division, University of Southern California, Los Angeles; 11 Department of Neurology, Vagelos College of Physicians and Surgeons, Columbia University, New York, New York; 12 Department of Epidemiology, Mailman School of Public Health, Columbia University, New York, New York; 13 Departments of Neurology and Public Health Sciences, University of Virginia, Charlottesville; 14 Department of Neurology, University of Arizona–Tucson; 15 Department of Neurology, McGovern Medical School at UTHealth, Houston, Texas; 16 Department of Neurology, University of Miami Miller School of Medicine, Miami, Florida; 17 Department of Neurology and Neurotherapeutics, University of Texas–Southwestern, Dallas; 18 Department of Neurology, University of Texas–San Antonio; 19 Department of Neurology, Medstar Georgetown University Hospital, Washington, DC; 20 Department of Neurology and Neurosurgery, University of Tennessee Health Sciences, Memphis; 21 Department of Neurology, Yale University, New Haven, Connecticut; 22 John P. Hussman Institute for Human Genomics, University of Miami Miller School of Medicine, Miami, Florida

## Abstract

**Importance:**

Black and Hispanic individuals have an increased risk of intracerebral hemorrhage (ICH) compared with their White counterparts, but no large studies of ICH have been conducted in these disproportionately affected populations.

**Objective:**

To examine the prevalence, odds, and population attributable risk (PAR) percentage for established and novel risk factors for ICH, stratified by ICH location and racial/ethnic group.

**Design, Setting, and Participants:**

The Ethnic/Racial Variations of Intracerebral Hemorrhage Study was a case-control study of ICH among 3000 Black, Hispanic, and White individuals who experienced spontaneous ICH (1000 cases in each group). Recruitment was conducted between September 2009 and July 2016 at 19 US sites comprising 42 hospitals. Control participants were identified through random digit dialing and were matched to case participants by age (±5 years), sex, race/ethnicity, and geographic area. Data analyses were conducted from January 2019 to May 2020.

**Main Outcomes and Measures:**

Case and control participants underwent a standardized interview, physical measurement for body mass index, and genotyping for the ɛ2 and ɛ4 alleles of *APOE*, the gene encoding apolipoprotein E. Prevalence, multivariable adjusted odds ratio (OR), and PAR percentage were calculated for each risk factor in the entire ICH population and stratified by racial/ethnic group and by lobar or nonlobar location.

**Results:**

There were 1000 Black patients (median [interquartile range (IQR)] age, 57 [50-65] years, 425 [42.5%] women), 1000 Hispanic patients (median [IQR] age, 58 [49-69] years; 373 [37.3%] women), and 1000 White patients (median [IQR] age, 71 [59-80] years; 437 [43.7%] women). The mean (SD) age of patients with ICH was significantly lower among Black and Hispanic patients compared with White patients (eg, lobar ICH: Black, 62.2 [15.2] years; Hispanic, 62.5 [15.7] years; White, 71.0 [13.3] years). More than half of all ICH in Black and Hispanic patients was associated with treated or untreated hypertension (PAR for treated hypertension, Black patients: 53.6%; 95% CI, 46.4%-59.8%; Hispanic patients: 46.5%; 95% CI, 40.6%-51.8%; untreated hypertension, Black patients: 45.5%; 95% CI, 39.%-51.1%; Hispanic patients: 42.7%; 95% CI, 37.6%-47.3%). Lack of health insurance also had a disproportionate association with the PAR percentage for ICH in Black and Hispanic patients (Black patients: 21.7%; 95% CI, 17.5%-25.7%; Hispanic patients: 30.2%; 95% CI, 26.1%-34.1%; White patients: 5.8%; 95% CI, 3.3%-8.2%). A high sleep apnea risk score was associated with both lobar (OR, 1.68; 95% CI, 1.36-2.06) and nonlobar (OR, 1.62; 95% CI, 1.37-1.91) ICH, and high cholesterol was inversely associated only with nonlobar ICH (OR, 0.60; 95% CI, 0.52-0.70); both had no interactions with race and ethnicity. In contrast to the association between the ɛ2 and ɛ4 alleles of* APOE *and ICH in White individuals (eg, presence of *APOE* ɛ2 allele: OR, 1.84; 95% CI, 1.34-2.52), *APOE* alleles were not associated with lobar ICH among Black or Hispanic individuals.

**Conclusions and Relevance:**

This study found sleep apnea as a novel risk factor for ICH. The results suggest a strong contribution from inadequately treated hypertension and lack of health insurance to the disproportionate burden and earlier onset of ICH in Black and Hispanic populations. These findings emphasize the importance of addressing modifiable risk factors and the social determinants of health to reduce health disparities.

## Key Points

**Question:**

Does the prevalence and burden of risk factors for lobar and nonlobar intracerebral hemorrhage (ICH) differ among Black, Hispanic, and White populations?

**Findings:**

In this case-control study of 3000 cases of ICH among Black, Hispanic, and White patients, the ɛ2 and ɛ4 alleles of* APOE,* the gene encoding apolipoprotein E, were associated with lobar ICH in White but not Black and Hispanic patients; hypertension was a risk factor for both lobar and nonlobar ICH in all groups; and the mean age for ICH among Black and Hispanic patients was more than 10 years younger than that of their White counterparts. Black and Hispanic patients had a higher attributable risk percentage for treated or untreated hypertension and lack of health insurance than White patients.

**Meaning:**

These findings suggest that potentially modifiable risk factors and social determinants of health are important contributors to the disproportionate ICH burden experienced by Black and Hispanic populations.

## Teaser

This case-control study examines the prevalence, odds, and population attributable risk for established and novel risk factors for intracerebral hemorrhage, stratified by intracerebral hemorrhage location and racial/ethnic group.

## Introduction

Intracerebral hemorrhage (ICH) is the most severe subtype of stroke, with a high rate of mortality and persistent disability among survivors.^1^ Compared with their White counterparts, Black and Hispanic individuals are at a higher risk of ICH, especially at younger ages.^2,3,4,5,6^ There are important gaps in our understanding of the risk factors for ICH among Black and Hispanic patients. Prior studies have been relatively small, with limited precision of the association of risk factors with ICH. We need precise estimates of risk factor prevalence and strength of association to determine the population-level impact of risk factors. Although prior studies have found stronger associations of amyloid angiopathy with lobar hemorrhage and of hypertension with nonlobar hemorrhage in largely White populations, little research has focused on the differences in risk factors by location in Black and Hispanic populations. The Ethnic/Racial Variations of Intracerebral Hemorrhage (ERICH) study was designed to address these gaps by conducting a large study with an equal number of Black, Hispanic, and White patients. In this article, we present the prevalence, odds ratio (OR), and population attributable risk (PAR) percentage findings for established and novel risk factors for ICH, stratified by ICH location, and we examine variation across racial/ethnic groups.

## Methods

### Source of Sample and Risk Factor Assessment

ERICH study methods have been described previously.^7,8^ In brief, ERICH was a multicenter, prospective, case-control study of risk factors for ICH. It was designed to recruit 1000 ICH case participants and 1000 control participants from non-Hispanic Black, Hispanic, and non-Hispanic White populations, for a total of 3000 case participants and 3000 control participants. Race/ethnicity was determined by self-report using federally mandated definitions.^9^ Participants were recruited from 19 US sites comprising 42 hospitals from September 2009 and July 2016. Control participants were identified through random digit dialing and were matched to cases by age (±5 years), sex, race/ethnicity, and geographic area. Inclusion criteria were as follows: aged 18 years or older; residency within 50 miles of the recruitment center or 100 miles for population centers with less than 1 million residents; Black, Hispanic, or White race/ethnicity; and, for case participants, a spontaneous ICH not attributable to hemorrhagic conversion of a cerebral infarction or structural vascular anomalies. All participating centers obtained institutional review board approval, and informed consent was obtained from all case and control participants or their legally authorized representative. This study followed the Strengthening the Reporting of Observational Studies in Epidemiology (STROBE) reporting guideline.

Computed tomography images on admission, as well as follow-up imaging during hospitalization, were reviewed. Data collected on case participants by each site included whether location of hemorrhage was lobar or nonlobar. Nonlobar hemorrhages included deep subcortical, brainstem, and cerebellar hemorrhages. Deidentified images in digital format were also centrally reviewed for eligibility and ICH location in a standardized fashion, masked to clinical evaluations. A panel reviewed all discrepancies for final adjudication.

Candidate risk factors were selected based on prior literature review and biological plausibility. All participants or designated proxies underwent a standardized interview, including questions on age, sex, race/ethnicity, treated or untreated hypertension, diabetes, ischemic stroke, chronic kidney disease, elevated cholesterol, high risk of obstructive sleep apnea (OSA) by the Berlin Questionnaire,^10^ antiplatelet use, anticoagulant use, cigarette smoking, alcohol use, cocaine or amphetamine use, and medical insurance status. Case and control participants had physical measurements for body mass index (BMI; calculated as weight in kilograms divided by height in meters squared) and were genotyped for polymorphisms of *APOE* (OMIM 107741)*,* the gene encoding apolipoprotein E.

An external validity check was performed to compare enrolled and nonenrolled patients during a 6-month period to ascertain the representativeness of the enrolled patients. Enrolled patients were slightly younger than nonenrolled patients (mean [SD] age, 62.0 [14.5] years vs 65.1 [14.5] years; *P* < .001), and there was a higher mortality in the nonenrolled patients vs enrolled patients (118 of 373 [31.6%] vs 334 of 3000 [11.1%]; *P* < .001), but there were no significant differences in medical record–ascertained risk factors.^11^

### Statistical Analysis

Differences across the 3 race/ethnicities in the distribution of age, sex, and ICH location were assessed using the Kruskal-Wallis test for age and χ^2^ tests for categorical variables. Differences across the 3 race/ethnicity groups in 2-level risk factors were assessed using logistic regression after adjusting for age and sex. Differences across the 3 race/ethnicity groups in the distributions of multilevel risk factors, smoking status, hypertension, BMI, and alcohol use were assessed by ordinal logistic regression. *APOE* risk allele status was modeled as 2 variables, ɛ2 and ɛ4; each variable was coded as 1 if the allele was present, with the reference category being homozygous ɛ3.^12^

Case-control association analyses stratified by race/ethnicity were performed for all ICH and stratified by lobar vs nonlobar location. In case-control analyses stratified by lobar and nonlobar location, the control participants for both ICH subtypes were included in the models for each subtype to enhance statistical power; otherwise, only the subtype-specific control participants were used. In these analyses, univariable logistic regression models for occurrence of ICH were first constructed using each of the risk factors. The multivariable model was constructed using all risk factors that were *P* < .20 in the univariable models, with backward elimination to retain only those factors that were *P* ≤ .05. In the race/ethnicity-stratified analyses, this procedure was repeated separately for each subgroup. PAR percentage for each risk factor stratified by race/ethnicity was calculated^13^ for all ICH using risk factor prevalence among control participants and the multivariable adjusted OR to account for the association of the other risk factors. For some risk factors and particularly multilevel risk factors, ORs included values less than 1, indicating a protective risk factor, resulting in negative PAR percentage values.

Interactions with race/ethnicity were sought by including all potential race/ethnicity interactions with the main effects. The main effects that were significant in any of the 3 racial/ethnic groups were included for the interaction models. All main effects were retained, and interaction effects were retained if they were significant at the *P* < .05 level after backward elimination. Two interaction models, 1 for each of lobar and nonlobar ICH, were constructed.

Missing data were not imputed. Statistical analysis was performed using SAS version 9.4 software (SAS Institute). Statistical significance was set at *P* < .05, and all hypothesis tests were 2-sided.

## Results

There were 1000 Black patients (median [interquartile range (IQR)] age, 57 [50-65] years, 425 [42.5%] women), 1000 Hispanic patients (median [IQR] age, 58 [49-69] years; 373 [37.3%] women), and 1000 White patients (median [IQR] age, 71 [59-80] years; 437 [43.7%] women) (age: *P* < .001; sex: *P* = .008) (Table 1). Black and Hispanic patients had a substantially lower proportion of lobar ICH compared with White patients (238 [23.8%] and 274 [27.4%] vs 420 [42.0%]; *P* < .001). Adjusted for age and sex, Black and Hispanic cases had a higher prevalence of prior history of ischemic stroke, chronic kidney disease, untreated hypertension, diabetes, heavy alcohol use, and cocaine or amphetamine use and a lower prevalence of history of hypercholesterolemia, anticoagulant use, and medical insurance than White patients. Black case participants had a higher rate of current smoking than Hispanic and White case participants. Hispanic cases participants had a lower proportion of *APOE* ɛ2 than Black or White case participants, whereas Black cases participants had a higher proportion of *APOE* ɛ4 than Hispanic or White case participants. BMI distributions and high OSA risk was similar across the 3 racial/ethnic groups.

**Table 1.** Distribution of Intracerebral Hemorrhage Cases by Age, Sex, Intracerebral Hemorrhage Location, and Risk Factors Across the 3 Racial and Ethnic Groups

Variable	Patients, No. (%)			*P* value
	Black (n = 1000)	Hispanic (n = 1000)	White (n = 1000)	
Age, median (IQR), y	57 (50-65)	58 (49-69)	71 (59-80)	<.001^a^
Sex				
Women	425 (42.5)	373 (37.3)	437 (43.7)	.008^b^
Men	575 (57.5)	627 (62.7)	563 (56.3)	
Location				
Lobar	238 (23.8)	274 (27.4)	420 (42.0)	<.001^b^
Deep	615 (61.5)	563 (56.3)	446 (44.6)	
Brainstem	63 (6.3)	56 (5.6)	38 (3.8)	
Cerebellum	73 (7.3)	90 (9.0)	73 (7.3)	
Pure IVH	11 (1.1)	17 (1.7)	23 (2.3)	
Age- and sex-adjusted comparisons				
Ischemic stroke history	99 (9.9)	99 (9.9)	87 (8.7)	.03
Chronic kidney disease	107 (10.7)	95 (9.5)	57 (5.7)	<.001
Hypertension				
None	135 (13.7)	225 (22.9)	278 (28.1)	<.001^c^
Treated	485 (49.2)	394 (40.1)	480 (48.6)	
Untreated	366 (37.1)	363 (37.0)	230 (23.3)	
Diabetes	240 (24.2)	308 (31.0)	210 (21.1)	<.001
High sleep apnea risk	415 (41.6)	408 (40.8)	356 (35.7)	.91
BMI				
<18.5	24 (2.5)	20 (2.0)	24 (2.5)	.29^c^
18.5 to <25	262 (27.3)	229 (23.3)	357 (37.0)	
25 to <30	282 (29.3)	385 (39.2)	304 (31.5)	
≥30	393 (40.9)	347 (35.4)	281 (29.1)	
High cholesterol	331 (35.0)	377 (40.3)	514 (53.8)	.002
Smoking				
Never	489 (49.4)	559 (56.2)	490 (49.3)	<.001^c^
Current	277 (28.0)	143 (14.4)	131 (13.2)	
Former	224 (22.6)	292 (29.4)	373 (37.5)	
Alcohol use^d^				.006^c^
None or rare	584 (60.3)	597 (61.2)	599 (60.6)	
Moderate	274 (28.3)	264 (27.1)	322 (32.6)	
Heavy	110 (11.4)	114 (11.7)	68 (6.9)	
Cocaine or amphetamine use	72 (7.2)	38 (3.8)	18 (1.8)	<.001
Antiplatelet use	32 (3.2)	37 (3.7)	67 (6.7)	.13
Anticoagulant use	49 (4.9)	66 (6.6)	142 (14.2)	.002
*APOE*				
Presence of *APOE* ɛ2 allele	197 (19.8)	72 (7.2)	172 (17.7)	<.001
Presence of *APOE* ɛ4 allele	390 (39.3)	215 (21.6)	290 (29.9)	<.001
Lack of medical insurance	332 (33.2)	387 (38.7)	112 (11.2)	<.001

Abbreviations: BMI, body mass index (calculated as weight in kilograms divided by height in meters squared); IQR, interquartile range; IVH, intraventricular hemorrhage.

^a^ *P* value by Kruskal-Wallis test.

^b^ *P* value by χ^2^ test.

^c^ Analysis by ordinal logistic regression.

^d^ None or rare use indicates less than 1 drink per month; moderate use, 1 drink per month to 4 drinks per day; heavy use, 5 or more drinks per day, per Chen et al,^8^ 2017.

The adjusted ORs for treated hypertension among Black, Hispanic, and White participants were 3.16 (95% CI, 2.36-4.25), 3.13 (95% CI, 2.39-4.11), and 1.74 (95% CI, 1.38-2.20), respectively. PAR percentages for treated hypertension in Black and Hispanic participants were 53.6% (95% CI, 46.4%-59.8%) and 46.5% (95% CI, 40.6%-51.8%), respectively, compared with 26.3% (95% CI, 17.8%-33.8%) in White participants (Table 2). Similarly, the PAR percentages for untreated hypertension were much higher in Black and Hispanic participants compared with White participants (Black: 45.5%; 95% CI, 39.4%-51.1%; Hispanic: 42.7%; 95% CI, 37.6%-47.3%; White: 22.1%; 95% CI, 17.3%-26.7%). High OSA risk was associated with a PAR percentage of 18.9% (95% CI, 12.7%-24.7%) in Black participants and 14.4% (95% CI, 9.0-19.5%) in White participants but did not achieve the threshold for model inclusion in Hispanic participants. Lack of medical insurance was associated with PAR percentages of 21.7% (95% CI, 17.%-25.7%), 30.2% (95% CI, 26.1%-34.1%), and 5.8% (95% CI, 3.3%-8.2%) in Black, Hispanic, and White participants, respectively. eTable 1, eTable 2, and eTable 3 in the Supplement show the univariable and multivariable odds ratios and *P* values for all variables in the original models without backward elimination for each racial/ethnic group.

**Table 2.** Prevalence of Risk Factors, Multivariable ORs, and PAR Percentages for Any Intracerebral Hemorrhage Among Control Participants, Stratified by Race/Ethnicity

Risk factor	Black participants (885 case; 979 control)^a^			Hispanic participants (895 case; 969 control)^a^				White participants (891 case; 990 control)^a^		
	Prevalence, No. (%)	OR (95% CI)^b^	PAR, % (95% CI)^b^	Prevalence, No. (%)	OR (95% CI)^b^	PAR, % (95% CI)^b^	Prevalence, No. (%)		OR (95% CI)^b^	PAR, % (95% CI)^b^
Ischemic stroke history	13 (1.3)	7.60 (4.05 to 14.25)	8.1 (6.0 to 10.1)	15 (1.5)	5.6 (3.15 to 10.21)	6.7 (4.5 to 8.9)	11 (1.1)		7.92 (4.08 to 15.36)	7.1 (5.1 to 9.2)
Chronic kidney disease	39 (4.0)	2.33 (1.51 to 3.59)	5.0 (2.5 to 7.5)	39 (4.0)	2.40 (1.55 to 3.74)	5.4 (2.9 to 7.7)	NA		NA	NA
Hypertension										
None	382 (39.0)	1 [Reference]	NA	511 (52.7)	1 [Reference]	NA	475 (48.0)		1 [Reference]	NA
Treated	522 (53.3)	3.16 (2.36 to 4.25)	53.6 (46.4 to 59.8)	395 (40.8)	3.13 (2.39 to 4.11)	46.5 (40.6 to 51.8)	476 (48.1)		1.74 (1.38 to 2.20)	26.3 (17.8 to 33.8)
Untreated	75 (7.7)	11.92 (8.37 to 16.96)	45.5 (39.4 to 51.1)	63 (6.5)	12.45 (8.80 to 17.63)	42.7 (37.6 to 47.3)	39 (3.9)		8.22 (5.51 to 12.25)	22.1 (17.3 to 26.7)
BMI										
<18.5	9 (0.9)	140 (0.55 to 3.59)	0.4 (−2.0 to 2.7)	2 (0.2)	8.00 (1.66 to 38.54)	1.4 (−0.6 to 3.4)	14 (1.4)		1.22 (0.58 to 2.56)	0.3 (−1.8 to 2.4)
18.5 to <25	170 (17.4)	1 [Reference]	NA	184 (19.0)	1 [Reference]	NA	317 (32.0)		1 [Reference]	NA
25 to <30	34 (34.1)	0.62 (0.46 to 0.85)	−14.7 (−28.3 to −2.6)	360 (37.2)	0.72 (0.54 to 0.97)	−11.5 (−26.1 to 1.3)	361 (36.5)		0.66 (0.52 to 0.85)	−14.0 (−24.7 to −4.2)
≥30	466 (47.6)	0.46 (0.33 to 0.63)	−35.0 (−54.0 to −18.2)	423 (43.7)	0.54 (0.40 to 0.73)	−24.9 (−41.6 to −10.2)	298 (30.1)		0.54 (0.41 to 0.71)	−16.1 (−26.3 to 06.8)
High sleep apnea risk	331 (33.8)	1.69 (1.31 to 2.18)	18.9 (12.7 to 24.7)	NA	NA	NA	234 (23.6)		1.71 (1.33 to 2.20)	14.4 (9.0 to 19.5)
High cholesterol	429 (43.8)	0.60 (0.47 to 0.77)	−21.0 (−30.2 to −12.4)	474 (48.9)	0.63 (0.50 to 0.80)	−22.1 (−32.7 to 12.5)	570 (57.6)		0.71 (0.57 to 0.89)	−19.8 (−32.6 to −8.2)
Smoking										
Never	457 (46.7)	1 [Reference]	NA	NA	NA	NA	NA		NA	NA
Former	277 (28.3)	0.67 (0.51 to 0.88)	−10.2 (−17.8 to −3.2)	NA	NA	NA	NA		NA	NA
Current	245 (25.0)	0.68 (−.51 to 0.90)	−8.8 (−16.4 to −1.7)	NA	NA	NA	NA		NA	NA
Alcohol use										
None or rare	564 (57.6)	1 [Reference]	NA	586 (60.5)	1 [Reference]	NA	440 (44.4)		1 [Reference]	NA
Moderate	375 (38.3)	0.76 (0.60 to 0.97)	−10.0 (017.9 to −2.8)	353 (36.4)	0.81 (0.64 to 1.03)	−7.5 (−14.7 to −0.7)	496 (50.1)		0.49 (0.40 to 0.61)	−34.2 (−45.7 to −23.7)
Heavy	40 (4.1)	2.08 (1.31 to 3.30)	4.2 (1.0 to 7.3)	30 (3.1)	3.10 (1.91 to 5.02)	6.1 (3.1 to 9.0)	54 (5.5)		0.82 (0.53 to 1.27)	−1.0 (−4.2 to 2.1)
Cocaine or amphetamine use	15 (1.5)	4.31 (2.22 to 8.38)	4.8 (3.0 to 6.7)	9 (0.9)	3.39 (1.46 to 7.87)	2.2 (0.8 to 3.6)	NA		NA	NA
Anticoagulant use	13 (1.3)	5.92 (3.00 to 11.70)	6.1 (4.5 to 7.8)	26 (2.7)	3.40 (2.04 to 5.68)	6.0 (4.1 to 8.0)	67 (6.8)		2.51 (1.79 to 3.51)	9.3 (6.3 to 12.2)
Presence of *APOE *ɛ4 allele	NA	NA	NA	NA	NA	NA	239 (24.1)		1.36 (1.08 to 1.71)	7.9 (2.7 to 12.9)
Lack of insurance	132 (13.5)	3.06 (2.31 to 4.04)	21.7 (17.5 to 25.7)	143 (14.8)	3.93 (2.99 to 5.17)	30.2 (26.1 to 34.1)	41 (4.1)		2.49 (1.60 to 3.85)	5.8 (3.3 to 8.2)

Abbreviations: BMI, body mass index (calculated as weight in kilograms divided by height in meters squared); NA, not applicable; OR, odds ratio; PAR, population attributable risk.

^a^ Numbers vary from 1000 because observations with missing values for any variable were excluded during the logistic regression.

^b^ The ORs and PAR percentages for each racial/ethnic group were adjusted only for the variables that were significant for that race/ethnic group.

For both lobar and nonlobar ICH, history of ischemic stroke, chronic kidney disease, hypertension, low BMI, OSA risk, cocaine or amphetamine use, anticoagulant use, and lack of medical insurance were associated with increased risk, and moderate alcohol use was associated with decreased risk (Table 3). For lobar ICH only, *APOE* ɛ2 and *APOE* ɛ4 were associated with increased risk. For nonlobar ICH only, heavy alcohol use was associated with increased risk, while high cholesterol and current and former smoking were associated with a lower risk. Diabetes and antiplatelet use were not associated with risk for ICH at either location. eTable 4 and eTable 5 in the Supplement show the univariable and multivariable ORs and *P* values for all variables in the original models without backward elimination.

**Table 3.** Multivariable ORs for Lobar and Nonlobar ICH

Risk factor	OR (95% CI)^a^	
	Lobar ICH (849 cases; 2929 controls)	Non-lobar ICH (1856 cases; 2941 controls)
Age	1.01 (1.00-1.02)	NA
Sex	1.44 (1.21-1.72)	NA
Race/ethnicity		
White	1 [Reference]	1 [Reference]
Black	0.50 (0.40-0.63)	0.80 (0.67-0.96)
Hispanic	0.68 (0.55-0.84)	0.84 (0.71-1.01)
Ischemic stroke history	5.50 (3.59-8.43)	7.85 (5.40-11.41)
Chronic kidney disease	1.53 (1.08-2.17)	1.92 (1.46-2.54)
Hypertension		
None	1 [Reference]	1 [Reference]
Treated	1.56 (1.27-1.93)	2.91 (2.43-3.48)
Untreated	5.61 (4.25-7.40)	13.40 (10.69-16.79)
BMI		
<18.5	1.61 (0.83-3.10)	1.78 (0.98-3.20)
18.5 to <25	1 [Reference]	1 s[Reference]
25 to <30	0.61 (0.49-0.75)	0.74 (0.62-0.89)
≥30	0.36 (0.28-0.46)	0.59 (0.48-0.72)
High sleep apnea risk	1.68 (1.36-2.06)	1.62 (1.37-1.91)
High cholesterol	NA	0.60 (0.52-0.70)
Smoking		
Never	NA	1 [Reference]
Former	NA	0.78 (0.66-0.91)
Current	NA	0.81 (0.66-1.00)
Alcohol use		
None or rare	1 [Reference]	1 [Reference]
Moderate	0.58 (0.48-0.70)	0.71 (0.61-0.82)
Heavy	1.42 (0.99-2.05)	1.92 (1.45-2.55)
Cocaine or amphetamine use	3.90 (2.06-7.39)	4.27 (2.55-7.16)
Anticoagulant use	2.81 (2.04-3.89)	3.06 (2.29-4.09)
*APOE*		
Presence of *APOE* ɛ2 allele	1.43 (1.14-1.80)	NA
Presence of *APOE* ɛ4 allele	1.41 (1.18-1.70)	NA
Lack of medical insurance	2.49 (1.92-3.23)	3.87 (3.19-4.70)

Abbreviations: BMI, body mass index (calculated as weight in kilograms divided by height in meters squared); ICH, intracerebral hemorrhage; NA, not applicable; OR, odds ratio.

^a^ The ORs for lobar and nonlobar ICH were adjusted only for the variables that were significant for that phenotype.

In each racial/ethnic group, history of ischemic stroke, hypertension, anticoagulant use, and lack of medical insurance were associated with increased risk of lobar ICH, whereas overweight or obesity were associated with decreased risk (Table 4). Moderate alcohol use was associated with decreased risk in Black and White participants, but the association between moderate alcohol use and decreased risk was not significant among Hispanic participants. The only significant interactions with race/ethnicity were for anticoagulant use and *APOE* ɛ4. Black patients (OR, 6.76; 95% CI, 3.01-15.19; *P* < .001) and, to a lesser extent, Hispanic patients (OR, 4.03; 95% CI, 2.12-7.65; *P* < .001) had significantly greater risk associated with anticoagulation use (*P *for interaction = .02). APOE ɛ4 was only associated with lobar ICH among White participants (OR, 1.84; 95% CI, 1.39-2.43; *P* < .001), not among Black or Hispanic participants (*P *for interaction = .02). An additional analysis was performed with lobar case and control participants aged 60 years and older, adjusting for age, sex, and history of hypertension, either treated or untreated. In this older age group, the ORs for *APOE* ɛ4 were 1.28 (95% CI, 0.84-1.95) for Black participants with 129 cases, 1.73 (95% CI, 1.12-2.66) for Hispanic participants with 152 cases, and 2.35 (95% CI, 1.75-3.15) for White participants with 326 cases. eTable 6, eTable 7, and eTable 8 in the Supplement provide the univariable and multivariable odds ratios and *P* values for all variables in the original models without backward elimination and show that the mean (SD) ages of lobar ICH for Black, Hispanic, and White patients were 62.2 (15.2) years, 62.5 (15.7) years, and 71.0 (13.3) years, respectively.

**Table 4.** Multivariable ORs for Lobar Intracerebral Hemorrhage, Stratified by Race^a^

Risk factor	Black participants (215 case; 983 control)		Hispanic participants (255 case; 973 control)		White participants (384 case; 990 control)		*P *for interaction
	OR (95% CI)	*P* value	OR (95% CI)	*P* value	OR (95% CI)	*P* value	
Age	NA	NA	1.02 (1.00-1.03)	.02	NA	NA	NA
Female	1.46 (1.04-2.05)	.03	1.95 (1.36-2.72)	<.001	NA	NA	NA
Ischemic stroke history	4.65 (2.12-10.20)	<.001	4.24 (1.99-9.06)	<.001	7.16 (3.47-14.78)	<.001	NA
Chronic kidney disease	2.56 (1.44-4.54)	.001	NA	NA	NA	NA	NA
Hypertension							
None	1 [Reference]	NA	1 [Reference]	NA	1 [Reference]	NA	NA
Treated	2.18 (1.41-3.37)	.001	1.84 (1.23-2.76)	.003	1.45 (1.09-1.94)	.01	NA
Untreated	7.02 (4.20-11.74)	<.001	6.85 (4.22-11.14)	<.001	5.25 (3.27-8.41)	<.001	NA
BMI							
<18.5	1.92 (0.65-5.71)	.24	6.64 (1.14-38.69)	.04	0.88 (0.31-2.46)	.80	NA
18.5 to <25	1 [Reference]	NA	1 [Reference]	NA	1 [Reference]	NA	NA
25 to <30	0.51 (0.32-0.79)	.003	0.55 (0.37-0.81)	.003	0.64 (0.47-0.87)	.005	NA
≥30	0.45 (0.30-0.68)	<.001	0.26 (0.16-0.42)	<.001	0.42 (0.29-0.62)	<.001	NA
High sleep apnea risk	NA	NA	1.66 (1.13-2.43)	<.01	1.73 (1.25-2.40)	.001	NA
Alcohol use							
None or rare	1 [Reference]	NA	1 [Reference]	NA	1 [Reference]	NA	NA
Moderate	0.58 (0.40-0.87)	.007	0.72 (0.50-1.05)	.09	0.50 (0.38-0.66)	<.001	NA
Heavy	1.70 (0.87-3.33)	.12	3.05 (1.54-6.04)	.001	0.77 (0.43-1.37)	.37	NA
Cocaine or amphetamine use	3.78 (1.54-9.29)	.004	6.13 (2.17-17.35)	.001	NA	NA	
Anticoagulant use	6.76 (3.01-15.19)	<.001	4.03 (2.12-7.65)	<.001	2.18 (1.43-3.34)	<.001	.02
Presence of *APOE* ɛ2 allele	NA	NA	NA	NA	1.84 (1.34-2.52)	<.001	NA
Presence of *APOE* ɛ4 allele^c^	NA	NA	NA	NA	1.84 (1.39-2.43)	<.001	.01
Lack of medical insurance	1.92 (1.24-3.00)	.004	3.06 (2.03-4.61)	<.001	2.61 (1.53-4.45)	<.001	NA

Abbreviations: BMI, body mass index (calculated as weight in kilograms divided by height in meters squared); NA, not applicable; OR, odds ratio.

^a^ The ORs for each racial/ethnic group were adjusted only for the variables that were significant for that group.

In each racial/ethnic group, history of ischemic stroke, hypertension, anticoagulant use, and lack of medical insurance were associated with increased risk of nonlobar ICH, while obesity and history of high cholesterol were associated with decreased risk (Table 5). Both former and current smoking were associated with lower risk of nonlobar ICH in Black participants only. The only significant interactions with race/ethnicity were for alcohol use, lack of medical insurance, and age. Heavy alcohol use and lack of medical insurance were associated with a higher risk in Black participants (heavy alcohol use: OR, 2.33; 95% CI, 1.42-3.82; lack of insurance: OR, 3.66; 95% CI, 2.71-4.94) and, particularly, in Hispanic participants (heavy alcohol use: OR, 3.98; 95% CI, 2.37-6.69; lack of insurance: OR, 4.85; 95% CI, 3.56-6.60) compared with White participants (alcohol use, *P *for interaction < .001; lack of insurance, *P *for interaction = .03). Although there was no association of age within racial/ethnic groups because of matching, there was an interaction with age. eTable 9, eTable 10, and eTable 11 in the Supplement provide the univariable and multivariable odds ratios and *P* values for all variables in the original models without backward elimination and show that the mean (SD) ages of nonlobar ICH for Black, Hispanic, and White patients were 56.7 (11.6) years, 57.6 (13.4) years, and 67.8 (14.1) years, respectively.

**Table 5.** Multivariable ORs for Nonlobar Intracerebral Hemorrhage, Stratified by Race^a^

Risk Factors	Black participants (679 case; 979 control)		Hispanic participants (653 case; 969 control)		White participants (516 case; 990 control)		*P *for interaction
	OR (95% CI)	*P* value	OR (95% CI)	*P* value	OR (95% CI)	*P* value	
Age	NA	NA	NA	NA	NA	NA	.01
Female	NA	NA	NA	NA	0.75 (0.58-0.97)	.03	NA
Ischemic stroke history	8.50 (4.44-16.27)	<.001	6.94 (3.77-12.79)	<.001	8.10 (3.97-16.54)	<.001	NA
Chronic kidney disease	2.08 (1.30-3.33)	.002	2.70 (1.68-4.34)	<.001	NA	NA	NA
Hypertension							
None	1 [Reference]	NA	1 [Reference]	NA	1 [Reference]	NA	NA
Treated	3.64 (2.60-5.08)	<.001	3.92 (2.85-5.41)	<.001	2.06 (1.53-2.78)	<.001	NA
Untreated	14.98 (10.19-22.01)	<.001	17.00 (11.60-24.91)	<.001	11.72 (7.48-18.36)	<.001	NA
Diabetes	NA	NA	NA	NA	1.39 (1.00-1.93)	.05	
BMI							
<18.5	0.90 (0.31-2.63)	.85	5.52 (1.13-27.05)	.04	1.68 (0.71-3.95)	.24	NA
18.5 to <25	1 [Reference]	NA	1 [Reference]	NA	1 [Reference]	NA	NA
25 to <30	0.68 (0.48-0.95)	.02	0.88 (0.63-1.23)	.46	0.65 (0.48-0.88)	.005	NA
≥30	0.50 (0.35-0.72)	<.001	0.71 (0.51-1.00)	.47	0.59 (0.42-0.83)	.002	NA
High sleep apnea risk	1.81 (1.37-2.40)	<.001	NA	NA	1.81 (1.34-2.44)	<.001	NA
High cholesterol	0.60 (0.46-0.79)	<.001	0.56 (0.43-0.73)	<.001	0.55 (0.42-0.72)	<.001	NA
Smoking							
Never	1 [Reference]	NA	NA	NA	NA	NA	NA
Former	0.62 (0.46-0.83)	.001	NA	NA	NA	NA	NA
Current	0.66 (0.48-0.90)	.009	NA	NA	NA	NA	NA
Alcohol use							
None or rare	1 [Reference]	NA	1 [Reference]	NA	1 [Reference]	NA	<.001
Moderate	0.87 (0.67-1.13)	.29	0.92 (0.70-1.22)	.57	0.47 (0.36-0.61)	<.001	
Heavy	2.33 (1.42-3.82)	.001	3.98 (2.37-6.69)	<.001	0.88 (0.52-1.47)	.61	
Cocaine or amphetamine use	4.91 (2.45-9.86)	<.001	3.07 (1.20-7.84)	.02	7.29 (1.47-36.24)	.02	NA
Anticoagulant use	5.41 (2.57-11.39)	<.001	2.75 (1.54-4.91)	.001	2.84 (1.93-4.16)	<.001	NA
Presence of APOE ɛ2 allele	NA	NA	NA	NA	0.63 (0.45-0.90)	.010	NA
Presence of APOE ɛ4 allele	NA	NA	NA	NA	NA	NA	NA
Lack of insurance	3.66 (2.71-4.94)	<.001	4.85 (3.56-6.60)	<.001	2.50 (1.50-4.15)	<.001	.03

Abbreviations: BMI, body mass index (calculated as weight in kilograms divided by height in meters squared); NA, not applicable; OR, odds ratio.

^a^ The odds ratios for each race/ethnic group were adjusted only for the variables that were significant for that race/ethnic group.

## Discussion

The ERICH study fills an important gap in our knowledge of risk factors for ICH in the United States. The strengths of this study are the large and equal sample sizes in each of the 3 racial/ethnic groups, control participants from the same populations as case participants, centralized neuroimaging review, careful phenotyping into lobar and nonlobar ICH, a standardized interview for established and novel risk factors, and external validity analyses. The major findings of this study were that among Black and Hispanic participants, *APOE* was not associated with lobar ICH, whereas hypertension remained a strong risk factor for this ICH subtype. More than half of all ICH among Black and Hispanic populations was attributable to hypertension. Compared with White patients, Black and Hispanic patients had ICH at a much younger age and had a higher PAR percentage for both treated and untreated hypertension and lack of health insurance.

Differences in both prevalence and strength of association contribute to the higher PAR percentages for treated and untreated hypertension among Black and Hispanic participants. The stronger associations of treated and untreated hypertension with ICH risk in Black and Hispanic participants contributed importantly to the higher PAR percentages for these conditions. The higher ORs for treated hypertension among Black and Hispanic participants compared with White participants (Black: 3.16; 95% CI, 2.36-4.25; Hispanic: 3.13; 95% CI, 2.39-4.11; White: 1.74; 95% CI, 1.38-2.20) could be due to a variety of factors. National data^14^ has shown that Black and Hispanic individuals were less likely to achieve target blood pressure goals during treatment than White individuals. However, Black patients were more likely to receive combination antihypertensive therapies, suggesting more difficult to control hypertension, whereas Hispanic patients were less likely to receive combination antihypertensive treatment than White patients, suggesting less adequate treatment.

Although hypercholesterolemia is associated with a higher risk of other cardiovascular disease, it has been found to be associated with a lower risk of ICH. The prospective Honolulu Heart Program reported a nonlinear inverse association between hypercholesterolemia and ICH, with increased risk only in the lowest quintile of cholesterol^15^; other prospective cohort studies^16,17^ had similar findings. A smaller study in a predominantly White population^18^ found that history of high cholesterol had an independent inverse association with nonlobar, but not lobar, ICH. The present study reports a similar finding and extends it to Black and Hispanic populations. In contrast, a recent mendelian randomization analysis in predominantly White populations found an inverse association of genetically determined low-density lipoprotein cholesterol with both nonlobar and lobar ICH, which was stronger for lobar ICH.^19^ Future research using mendelian randomization methods are needed to confirm the findings in White populations and determine whether a similar association is present in Black and Hispanic populations.

We found limited evidence for an association of diabetes or smoking with ICH. Despite the higher prevalence of diabetes among Black and Hispanic participants, diabetes was only associated with nonlobar ICH among White participants. Smoking was inversely associated with nonlobar ICH overall, but this association was only statistically significant among Black participants. In view of prospective studies^20,21^ showing that smoking was associated with an increased risk of ICH in predominantly White populations, it is possible that competing risks explain our findings, eg, Black individuals who smoke may die of cardiac disease or cancer and be selectively removed.

We found an inverse association of BMI with both lobar and nonlobar ICH across all racial/ethnic groups. In contrast, a case-control study of a predominantly White population^22^ found that extremes of BMI were associated with an increased risk of deep, but not lobar, ICH. Findings from prospective studies are mixed with some^23,24,25^ but not all^26,27^ studies showing an increased risk of ICH with very low or high BMI. Further prospective studies with phenotyping into lobar and nonlobar ICH or studies using mendelian randomization methods are needed.

A prior study reported a high prevalence of OSA risk based on the Berlin Questionnaire among patients with ICH.^28^ Our case-control finding of an association of OSA, a modifiable risk factor, with both lobar and nonlobar ICH using the Berlin Questionnaire, even after adjustment for BMI and history of hypertension, is a novel finding and will require replication. A prior meta-analysis of 10 cohort studies of OSA and stroke^29^ found a 2-fold increased risk of stroke but did not report results specifically for ICH. A potential mechanism for this association is increased sympathetic neural activity during OSA with higher blood pressure during sleep.^30^

Once stratified by location, risk factors for ICH were largely similar by racial/ethnic group. Notably, we did not identify a hypertension × race/ethnicity interaction. However, our analysis did identify several risk factors that did have interactions by race/ethnicity. For lobar ICH, White participants had significantly greater risk associated with *APOE* ɛ4, while Black participants had significantly greater risk associated with anticoagulation use. A previous multivariable analysis from the ERICH study^12^ found that the association of *APOE* ɛ4 with lobar ICH was specific for White populations. In contrast, a subsequent analysis of the ERICH data with propensity score matching for hypertension and adjusting only for age and sex found *APOE* ɛ4 to be associated with lobar ICH in Hispanic populations as well.^31^ When restricted to case and control participants with lobar ICH aged 60 years and older, we found *APOE* ɛ4 to be associated with ICH in Hispanic but not Black participants. Despite the size of our study, due to the smaller proportion of lobar ICH and the younger age of onset, we had limited statistical power to examine this association in older Black participants. Of note, there have been similar race/ethnicity findings for the association of *APOE* ɛ4 with Alzheimer disease.^32^ Among White and Hispanic patients, both homozygous and heterozygous *APOE* ɛ4 were associated with Alzheimer disease, whereas among Black participants, this association was only present for homozygous *APOE* ɛ4. For Alzheimer disease, there is evidence that African ancestry–specific genetic factors near *APOE* account for this difference.^33^

Few studies have addressed the differential risk of anticoagulation-associated bleeding by race/ethnicity. Prior analysis of Medicare-eligible patients receiving dialysis found that Black and Hispanic individuals had a higher risk of hemorrhagic stroke, but this was not adjusted for other potential confounders.^34^ Among users of warfarin, there is evidence from the Veterans Administration (VA) Health Care System that Black and Hispanic patients had more gaps in monitoring of longer than 55 days.^35^ Similarly, a study from a non-VA outpatient registry^36^ found that both Black and Hispanic patients had a lower proportion of time in the therapeutic range compared with White patients.

For nonlobar ICH, there was a significant interaction between race/ethnicity and the association of heavy alcohol use with ICH risk. Hispanic and Black participants had increased risk associated with heavy alcohol use, whereas White participants did not. A prior analysis from the ERICH study reported similar findings and noted that there was no interaction with binge drinking,^8^ Given that prior research among White patients has supported an association of heavy alcohol use and ICH,^37^ it is possible that differential reporting by race may have contributed to our findings.

It is noteworthy that lack of medical insurance was strongly associated with both lobar and nonlobar ICH risk in each race/ethnicity group, even after adjustment for many other factors. Lack of medical insurance was associated with a similar degree of risk as cocaine, amphetamine, or anticoagulation use in each race/ethnic group but was associated with a much higher PAR percentage in Black and Hispanic participants.

### Limitations

This study has limitations. The primary limitation of this observational study is the case-control design with the inherent potential for bias due to competing risks, differential recall, and unrecognized confounding. Selection bias associated with race/ethnicity in the recruitment of case and control participants is also a potential source of bias. The lower mortality of recruited vs screened patients may have influenced findings, although there was no evidence for differences in medical record–ascertained risk factors. The results may not be generalizable to non-US populations.

## Conclusions

This study of risk factors for lobar and nonlobar ICH in Black, Hispanic, and White individuals identified OSA as a novel risk factor for ICH and found inadequately treated hypertension and lack of health insurance were associated with the disproportionate burden of ICH among Black and Hispanic individuals. Remarkably, Black and Hispanic patients had an age of onset for ICH more than 10 years earlier than their White counterparts. These findings emphasize the importance of addressing modifiable risk factors and the social determinants of health to reduce health disparities.

## Notes

Supplement. **eTable 1. **Black Case and Control Participants: Univariable and Multivariable Odds Ratios and *P* Values for All Variables in the Original Model Without Backwards Elimination **eTable 2.** Hispanic Case and Control Participants: Univariable and Multivariable Odds Ratios and *P* Values for All Variables in the Original Model Without Backwards Elimination **eTable 3. **White Case and Control Participants: Univariable and Multivariable Odds Ratios and *P* Values for All Variables in the Original Model Without Backwards Elimination **eTable 4. **Lobar Intracerebral Hemorrhage: Univariable and Multivariable Odds Ratios and *P* Values for All Variables in the Original Model Without Backwards Elimination **eTable 5. **Nonlobar Intracerebral Hemorrhage: Univariable and Multivariable Odds Ratios and *P* Values for All Variables in the Original Model Without Backwards Elimination **eTable 6. **Lobar Intracerebral Hemorrhage, Black Subgroup, Univariable and Multivariable Odds Ratios and *P* Values for All Variables in the Original Model Without Backwards Elimination **eTable 7. **Lobar Intracerebral Hemorrhage, Hispanic Subgroup, Univariable and Multivariable Odds Ratios and *P* Values for All Variables in the Original Model Without Backwards Elimination **eTable 8.** Lobar Intracerebral Hemorrhage, White Subgroup, Univariable and Multivariable Odds Ratios and *P* Values for All Variables in the Original Model Without Backwards Elimination **eTable 9. **Nonlobar Intracerebral Hemorrhage, Black Subgroup, Univariable and Multivariable Odds Ratios and *P* Values for All Variables in the Original Model Without Backwards Elimination **eTable 10. **Nonlobar Intracerebral Hemorrhage, Hispanic Subgroup, Univariable and Multivariable Odds Ratios and *P* Values for All Variables in the Original Model Without Backwards Elimination **eTable 11. **Nonlobar Intracerebral Hemorrhage, White Subgroup, Univariable and Multivariable Odds Ratios and *P* Values for All Variables in the Original Model Without Backwards Elimination
