# Supplementary material for: Ethnic and Racial Variation in Intracerebral Hemorrhage Risk Factors and Risk Factor Burden
Source: JAMA Netw Open. 2021 Aug 23;4(8):e2121921. doi: 10.1001/jamanetworkopen.2021.21921 (PMC8383133; doi:10.1001/jamanetworkopen.2021.21921)
Supplement: Supplement. — eTable 1. Black Case and Control Participants: Univariable and Multivariable Odds Ratios and P Values for All Variables in the Original Model Without Backwards Elimination eTable 2. Hispanic Case and Control Participants: Univariable and Multivariable Odds Ratios and P Values for All Variables in the Original Model Without Backwards Elimination eTable 3. White Case and Control Participants: Univariable and Multivariable Odds Ratios and P Values for All Variables in the Original Model Without Backwards Elimination eTable 4. Lobar Intracerebral Hemorrhage: Univariable and Multivariable Odds Ratios and P Values for All Variables in the Original Model Without Backwards Elimination eTable 5. Nonlobar Intracerebral Hemorrhage: Univariable and Multivariable Odds Ratios and P Values for All Variables in the Original Model Without Backwards Elimination eTable 6. Lobar Intracerebral Hemorrhage, Black Subgroup, Univariable and Multivariable Odds Ratios and P Values for All Variables in the Original Model Without Backwards Elimination eTable 7. Lobar Intracerebral Hemorrhage, Hispanic Subgroup, Univariable and Multivariable Odds Ratios and P Values for All Variables in the Original Model Without Backwards Elimination eTable 8. Lobar Intracerebral Hemorrhage, White Subgroup, Univariable and Multivariable Odds Ratios and P Values for All Variables in the Original Model Without Backwards Elimination eTable 9. Nonlobar Intracerebral Hemorrhage, Black Subgroup, Univariable and Multivariable Odds Ratios and P Values for All Variables in the Original Model Without Backwards Elimination eTable 10. Nonlobar Intracerebral Hemorrhage, Hispanic Subgroup, Univariable and Multivariable Odds Ratios and P Values for All Variables in the Original Model Without Backwards Elimination eTable 11. Nonlobar Intracerebral Hemorrhage, White Subgroup, Univariable and Multivariable Odds Ratios and P Values for All Variables in the Original Model Without Backwards Elimination [file jamanetwopen-e2121921-s001.pdf]

Supplemental Online Content

Kittner SJ, Sekar P, Comeau ME, et al. Ethnic and racial variation in intracerebral hemorrhage risk factors and risk factor burden. *JAMA Netw Open.* 2021;4(8):e2121921. doi:10.1001/jamanetworkopen.2021.21921

eTable 1. Black Case and Control Participants: Univariable and Multivariable Odds Ratios and *P* Values for All Variables in the Original Model Without Backwards Elimination

eTable 2. Hispanic Case and Control Participants: Univariable and Multivariable Odds Ratios and *P* Values for All Variables in the Original Model Without Backwards Elimination

eTable 3. White Case and Control Participants: Univariable and Multivariable Odds Ratios and *P* Values for All Variables in the Original Model Without Backwards Elimination

eTable 4. Lobar Intracerebral Hemorrhage: Univariable and Multivariable Odds Ratios and *P* Values for All Variables in the Original Model Without Backwards Elimination

eTable 5. Nonlobar Intracerebral Hemorrhage: Univariable and Multivariable Odds Ratios and *P* Values for All Variables in the Original Model Without Backwards Elimination

eTable 6. Lobar Intracerebral Hemorrhage, Black Subgroup, Univariable and Multivariable Odds Ratios and *P* Values for All Variables in the Original Model Without Backwards Elimination

eTable 7. Lobar Intracerebral Hemorrhage, Hispanic Subgroup, Univariable and Multivariable Odds Ratios and *P* Values for All Variables in the Original Model Without Backwards Elimination

eTable 8. Lobar Intracerebral Hemorrhage, White Subgroup, Univariable and Multivariable Odds Ratios and *P* Values for All Variables in the Original Model Without Backwards Elimination

eTable 9. Nonlobar Intracerebral Hemorrhage, Black Subgroup, Univariable and Multivariable Odds Ratios and *P* Values for All Variables in the Original Model Without Backwards Elimination

eTable 10. Nonlobar Intracerebral Hemorrhage, Hispanic Subgroup, Univariable and Multivariable Odds Ratios and *P* Values for All Variables in the Original Model Without Backwards Elimination

eTable 11. Nonlobar Intracerebral Hemorrhage, White Subgroup, Univariable and Multivariable Odds Ratios and *P* Values for All Variables in the Original Model Without Backwards Elimination

This supplemental material has been provided by the authors to give readers additional information about their work.

© 2021 Kittner SJ et al. *JAMA Network Open.*

eTable 1. Black Case and Control Participants: Univariable (Univ) and Multivariable (MV) Odds Ratios and P Values for All Variables in the Original Model Without Backwards Elimination

	Cases: N=999[a]	Controls: N=1000	Univ OR (CI)	p-Value	Numbers Missing		MV OR (CI)[b]	MV p-Value
	No. (%)	No. (%)			Cases	Control		
Age: Mean (SD)	58.0 (12.7)	58.0 (12.3)	1.00 (0.99, 1.01)	0.9743	0	0	1.00 (0.99, 1.01)	0.8806
Females	424 (42.4)	425 (42.5)	1.00 (0.84, 1.19)	0.9792	0	0	1.06 (0.84, 1.33)	0.6362
Ischemic History	99 (9.9)	14 (1.4)	7.75 (4.40, 13.66)	<0.0001	0	0	7.36 (3.90, 13.87)	<0.0001
Chronic Kidney Disease	107 (10.7)	39 (3.9)	2.96 (2.03, 4.31)	<0.0001	0	0	2.28 (1.48, 3.53)	0.0002
Hypertension:					13	1		
No Hypertension	135 (13.7)	394 (39.4)	Reference				Reference	
Treated Hypertension	485 (49.2)	530 (53.0)	2.67 (2.12, 3.36)	<0.0001			3.16 (2.33, 4.28)	<0.0001
Untreated Hypertension	366 (37.1)	75 (7.5)	14.24 (10.38, 19.53)	<0.0001			11.73 (8.21, 16.78)	<0.0001
Diabetes	240 (24.2)	223 (22.3)	1.11 (0.90, 1.37)	0.3172	8	1	1.06 (0.80, 1.40)	0.6642
BMI:					38	0		
<18.5	24 (2.5)	9 (0.9)	1.77 (0.80, 3.90)	0.1567			1.41 (0.55, 3.65)	0.4735
18.5 to <25	262 (27.3)	174 (17.4)	Reference				Reference	
25 to <30	282 (29.3)	343 (34.3)	0.55 (0.43, 0.70)	<0.0001			0.63 (0.46, 0.86)	0.0032
>=30	393 (40.9)	474 (47.4)	0.55 (0.44, 0.70)	<0.0001			0.46 (0.33, 0.64)	<0.0001
Sleep Apnea Risk	415 (41.6)	333 (33.3)	1.43 (1.19, 1.71)	0.0001	2	0	1.66 (1.28, 2.16)	0.0001
High Cholesterol	331 (35.0)	435 (43.7)	0.69 (0.58, 0.83)	<0.0001	53	4	0.60 (0.46, 0.77)	<0.0001
Smoking:					9	0		
Current Smoking	277 (28.0)	252 (25.2)	1.04 (0.84, 1.29)	0.6833			0.68 (0.51, 0.90)	0.0071
Former smoking	224 (22.6)	283 (28.3)	0.75 (0.61, 0.94)	0.0101			0.67 (0.51, 0.88)	0.0036
Never Smoking	489 (49.4)	465 (46.5)	Reference				Reference	
Alcohol Use:					31	16		
No/Rare use	584 (60.3)	566 (57.5)	Reference				Reference	
Moderate Use	274 (28.3)	378 (38.4)	0.70 (0.58, 0.85)	0.0004			0.76 (0.59, 0.97)	0.0265
Heavy Use	110 (11.4)	40 (4.1)	2.66 (1.82, 3.90)	<0.0001			2.09 (1.31, 3.30)	0.0020
Cocaine/Amphetamine Use	72 (7.2)	16 (1.6)	4.78 (2.76, 8.27)	<0.0001	0	0	4.38 (2.25, 8.54)	<0.0001
Antiplatelet use	32 (3.2)	24 (2.4)	1.35 (0.79, 2.30)	0.2782	0	0	1.13 (0.59, 2.17)	0.7202
Anticoagulant use	49 (4.9)	14 (1.4)	3.63 (1.99, 6.62)	<0.0001	0	0	5.85 (2.95, 11.60)	<0.0001
APOE: Presence of 2	197 (19.8)	193 (19.5)	1.02 (0.82, 1.27)	0.8647	6	12	0.93 (0.70, 1.23)	0.603

© 2021 Kittner SJ et al. *JAMA Network Open.*

Table S1. Black Cases and Controls: Univariable (Univ) and Multivariable (MV) Odds Ratios and P-Values for all Variables in the Original Model without Backwards Elimination (continued)

	Cases: N=999	Controls: N=1000	Univ OR (CI)	p-Value	Numbers Missing		MV OR (CI)	MV p-Value
	No. (%)	No.(%)			Cases	Controls		
APOE: Presence of 4	390 (39.3)	378 (38.3)	1.04 (0.87, 1.25)	0.6427	6	12	0.96 (0.76, 1.20)	0.7085
Lack of Insurance	332 (33.2)	137 (13.7)	3.14 (2.51, 3.92)	<0.0001	0	0	3.01 (2.26, 4.01)	<0.0001

Abbreviations: OR, Odds Ratio; CI, Confidence Interval; SD, Standard Deviation; BMI, Body Mass Index; APOE, Apolipoprotein E.

[a] One case was not interviewed and is not included in this table. [b] Multivariable model included 877 cases and 968 controls.

© 2021 Kittner SJ et al. *JAMA Network Open.*

eTable 2. Hispanic Case and Control Participants: Univariable (Univ) and Multivariable (MV) Odds Ratios and P Values for All Variables in the Original Model Without Backwards Elimination

	Cases: N=1000	Controls: N=980[a]	Univ OR (CI)	p-Value	Numbers Missing		MV OR (CI)[b]	MV p-Value
					Cases	Control		
Age: Mean (SD)	58.9 (14.3)	58.2 (14.6)	1.00 (1.00, 1.01)	0.2477	0	0	1.01 (1.00, 1.02)	0.0251
Females	373 (37.3)	366 (37.4)	1.00 (0.83, 1.20)	0.9828	0	0	1.34 (1.05, 1.70)	0.0185
Ischemic History	99 (9.9)	15 (1.5)	7.06 (4.07, 12.24)	<0.0001	0	0	5.80 (3.18, 10.57)	<0.0001
Chronic Kidney Disease	95 (9.5)	39 (4.0)	2.53 (1.72, 3.72)	<0.0001	0	0	2.48 (1.57, 3.90)	<0.0001
Hypertension:					18	1		
No Hypertension	225 (22.9)	514 (52.5)	Reference				Reference	
Treated Hypertension	394 (40.1)	402 (41.1)	2.24 (1.82, 2.76)	<0.0001			2.80 (2.08, 3.77)	<0.0001
Untreated Hypertension	363 (37.0)	63 (6.4)	13.16 (9.65, 17.94)	<0.0001			11.78 (8.20, 16.90)	<0.0001
Diabetes	308 (31.0)	279 (28.5)	1.13 (0.93, 1.37)	0.2217	6	0	1.03 (0.79, 1.35)	0.8136
BMI:					19	3		
<18.5	20 (2.0)	2 (0.2)	8.08 (1.86, 35.01)	0.0052			8.27 (1.70, 40.14)	0.0087
18.5 to <25	229 (23.3)	185 (18.9)	Reference				Reference	
25 to <30	385 (39.2)	362 (37.0)	0.86 (0.68, 1.09)	0.2173			0.79 (0.58, 1.06)	0.1178
>=30	347 (35.4)	428 (43.8)	0.66 (0.52, 0.83)	0.0005			0.54 (0.39, 0.75)	0.0003
Sleep Apnea Risk	408 (40.8)	322 (32.9)	1.41 (1.17, 1.70)	0.0002	1	0	1.28 (0.98, 1.67)	0.0741
High Cholesterol	377 (40.3)	479 (49.1)	0.70 (0.58, 0.84)	0.0001	65	4	0.59 (0.46, 0.76)	<0.0001
Smoking:					6	0		
Current Smoking	143 (14.4)	113 (11.5)	1.24 (0.95, 1.63)	0.1191			0.92 (0.65, 1.31)	0.6434
Former smoking	292 (29.4)	318 (32.4)	0.90 (0.74, 1.10)	0.3057			0.90 (0.70, 1.16)	0.4203
Never Smoking	559 (56.2)	549 (56.0)	Reference				Reference	

© 2021 Kittner SJ et al. *JAMA Network Open.*

eTable 2. Hispanic Case and Control Participants: Univariable (Univ) and Multivariable (MV) Odds Ratios and P Values for All Variables in the Original Model Without Backwards Elimination (continued)								
	Cases: N=1000	Controls: N=980	Univ OR (CI)	p-Value	Numbers Missing		MV OR (CI)	MV p-Value
					Cases	Control		
Alcohol Use					25	3		
No/Rare use	597 (61.2)	592 (60.6)	Reference				Reference	
Moderate Use	264 (27.1)	355 (36.3)	0.74 (0.61, 0.90)	0.0023			0.90 (0.70, 1.17)	0.4365
Heavy Use	114 (11.7)	30 (3.1)	3.77 (2.48, 5.72)	<0.0001			3.84 (2.31, 6.38)	<0.0001
Cocaine/Amphetamine Use	38 (3.8)	9 (0.9)	4.26 (2.05, 8.86)	0.0001	0	0	3.78 (1.62, 8.86)	0.0022
Antiplatelet use	37 (3.7)	36 (3.7)	1.01 (0.63, 1.61)	0.9750	0	0	1.04 (0.58, 1.84)	0.9078
Anticoagulant use	66 (6.6)	26 (2.6)	2.59 (1.63, 4.12)	<0.0001	0	0	3.30 (1.96, 5.57)	<0.0001
APOE: Presence of 2	72 (7.2)	76 (7.8)	0.92 (0.66, 1.29)	0.6289	5	7	0.75 (0.49, 1.15)	0.1892
APOE: Presence of 4	215 (21.6)	199 (20.4)	1.07 (0.86, 1.33)	0.5296	5	7	0.96 (0.73, 1.25)	0.7406
Lack of Insurance	387 (38.7)	146 (14.9)	3.61 (2.90, 4.48)	<0.0001	0	0	4.43 (3.32, 5.91)	<0.0001

Abbreviations: OR, Odds Ratio; CI, Confidence Interval; SD, Standard Deviation; BMI, Body Mass Index; APOE, Apolipoprotein E.
[a] 20 controls were not interviewed and are not included in this table. [b] Multivariable model included 962 cases and 884 controls.

© 2021 Kittner SJ et al. *JAMA Network Open.*

eTable 3. White Case and Control Participants: Univariable (Univ) and Multivariable (MV) Odds Ratios and P Values for All Variables in the Original Model Without Backwards Elimination

	Cases: N=999 [a]	Controls: N=1000	Univ OR (CI)	p-Value	Numbers Missing		MV OR (CI) [b]	MV p-Value
					Cases	Control		
Age: Mean (SD)	69.1 (13.9)	68.6 (13.2)	1.00 (1.00, 1.01)	0.3866	0	0	1.00 (0.99, 1.01)	0.8713
Females	437 (43.7)	442 (44.2)	0.98 (0.82, 1.17)	0.8372	0	0	0.92 (0.75, 1.14)	0.4664
Ischemic History	87 (8.7)	11 (1.1)	8.58 (4.55, 16.16)	<0.0001	0	0	7.40 (3.81, 14.39)	<0.0001
Chronic Kidney Disease	57 (5.7)	45 (4.5)	1.28 (0.86, 1.92)	0.2216	0	0	1.06 (0.67, 1.67)	0.8054
Hypertension:					11	0		
No Hypertension	278 (28.1)	481 (48.1)	Reference				Reference	
Treated Hypertension	480 (48.6)	480 (48.0)	1.73 (1.42, 2.10)	<0.0001			1.69 (1.32, 2.16)	<0.0001
Untreated Hypertension	230 (23.3)	39 (3.9)	10.20 (7.04, 14.77)	<0.0001			8.04 (5.38, 12.03)	<0.0001
Diabetes	210 (21.1)	161 (16.1)	1.39 (1.11, 1.75)	0.0042	4	0	1.22 (0.92, 1.62)	0.1619
BMI:					33	4		
<18.5	24 (2.5)	14 (1.4)	1.53 (0.78, 3.00)	0.2199			1.21 (0.56, 2.57)	0.6279
18.5 to <25	357 (37.0)	318 (31.9)	Reference				Reference	
25 to <30	304 (31.5)	363 (36.4)	0.75 (0.60, 0.92)	0.0074			0.64 (0.50, 0.82)	0.0005
>=30	281 (29.1)	301 (30.2)	0.83 (0.67, 1.04)	0.1034			0.52 (0.39, 0.70)	<0.0001
Sleep Apnea Risk	356 (35.7)	237 (23.7)	1.78 (1.47, 2.17)	<0.0001	1	0	1.71 (1.32, 2.20)	<0.0001
High Cholesterol	514 (53.8)	573 (57.3)	0.87 (0.72, 1.04)	0.1160	43	0	0.68 (0.54, 0.85)	0.0009
Smoking:					5	1		
Current Smoking	131 (13.2)	91 (9.1)	1.45 (1.08, 1.95)	0.0134			0.98 (0.68, 1.42)	0.9311
Former smoking	373 (37.5)	414 (41.4)	0.91 (0.75, 1.10)	0.3151			0.93 (0.75, 1.16)	0.5119

© 2021 Kittner SJ et al. *JAMA Network Open.*

eTable 3. White Case and Control Participants: Univariable (Univ) and Multivariable (MV) Odds Ratios and P Values for All Variables in the Original Model Without Backwards Elimination(continued)								
	Cases: N=999	Controls: N=1000			Numbers Missing		MV OR (CI)	MV p-Value
					Cases	Control		
Smoking (continued):<br><br>Never Smoking	490 (49.3)	494 (49.4)	Reference				Reference	
Alcohol Use:					10	3		
No/Rare use	599 (60.6)	442 (44.3)	Reference				Reference	
Moderate Use	322 (32.6)	501 (50.2)	0.47 (0.39, 0.57)	<0.0001			0.49 (0.39, 0.61)	<0.0001
Heavy Use	68 (6.9)	54 (5.4)	0.93 (0.64, 1.36)	0.7033			0.81 (0.51, 1.27)	0.3553
Cocaine/Amphetamine Use	18 (1.8)	2 (0.2)	9.16 (2.12, 39.56)	0.0030	0	0	4.59 (0.94, 22.52)	0.0602
Antiplatelet use	67 (6.7)	52 (5.2)	1.31 (0.90, 1.90)	0.1556	0	0	1.50 (0.98, 2.32)	0.0644
Anticoagulant use	142 (14.2)	67 (6.7)	2.31 (1.70, 3.13)	<0.0001	0	0	2.58 (1.83, 3.64)	<0.0001
APOE: Presence of 2	172 (17.7)	173 (17.4)	1.02 (0.81, 1.29)	0.8329	28	3	1.06 (0.81, 1.39)	0.6452
APOE: Presence of 4	290 (29.9)	240 (24.1)	1.34 (1.10, 1.64)	0.0038	28	3	1.37 (1.09, 1.73)	0.0075
Lack of Insurance	112 (11.2)	41 (4.1)	2.95 (2.04, 4.27)	<0.0001	0	0	2.38 (1.51, 3.76)	0.0002

Abbreviations: OR, Odds Ratio; CI, Confidence Interval; SD, Standard Deviation; BMI, Body Mass Index; APOE, Apolipoprotein E.

[a] One case was not interviewed and is not included in this table. [b] Multivariable model included 886 cases and 990 controls.

© 2021 Kittner SJ et al. *JAMA Network Open.*

eTable 4. Lobar Intracerebral Hemorrhage: Univariable (Univ) and Multivariable (MV) Odds Ratios and P Values for All Variables in the Original Model Without Backwards Elimination

	Cases: N=932	Controls: N=2980 [a]	Univar OR(CI)	p-Value	Numbers Missing		MV OR (CI) [b]	MV p-Value
	No. (%)				Cases	Control		
Age: Mean (SD)	66.2 (15.1)	61.6 (14.3)	1.02 (1.02, 1.03)	<0.0001	0	0	1.01 (1.00, 1.02)	0.0118
Race:				<0.0001	0	0		
Black	238 (25.5)	1000 (33.6)	0.57 (0.47, 0.68)	<0.0001			0.50 (0.40, 0.64)	<0.0001
Hispanic	274 (29.4)	980 (32.9)	0.67 (0.56, 0.79)	<0.0001			0.66 (0.53, 0.82)	0.0002
White	420 (45.1)	1000 (33.6)	Reference				Reference	
Females	457 (49.0)	1233 (41.4)	1.36 (1.18, 1.58)	<0.0001	0	0	1.42 (1.18, 1.70)	0.0001
Ischemic History	81 (8.7)	40 (1.3)	6.99 (4.75, 10.29)	<0.0001	0	0	5.02 (3.22, 7.81)	<0.0001
Chronic Kidney Disease	72 (7.7)	123 (4.1)	1.94 (1.44, 2.63)	<0.0001	0	0	1.60 (1.13, 2.26)	0.0085
Hypertension:				<0.0001	15	2		
No Hypertension	257 (28.0)	1389 (46.6)	Reference				Reference	
Treated Hypertension	448 (48.9)	1412 (47.4)	1.72 (1.45, 2.03)	<0.0001			1.62 (1.30, 2.02)	<0.0001
Untreated Hypertension	212 (23.1)	177 (5.9)	6.47 (5.09, 8.23)	<0.0001			5.36 (4.03, 7.13)	<0.0001
Diabetes	223 (24.1)	663 (22.3)	1.11 (0.93, 1.32)	0.2338	8	1	1.06 (0.85, 1.33)	0.5889
BMI:				<0.0001	39	7		
<18.5	23 (2.6)	25 (0.8)	1.97 (1.10, 3.53)	0.0223			1.62 (0.83, 3.13)	0.1564
18.5 to <25	316 (35.4)	677 (22.8)	Reference				Reference	
25 to <30	300 (33.6)	1068 (35.9)	0.60 (0.50, 0.72)	<0.0001			0.61 (0.49, 0.76)	<0.0001
>=30	254 (28.4)	1203 (40.5)	0.45 (0.37, 0.55)	<0.0001			0.37 (0.29, 0.48)	<0.0001
Sleep Apnea Risk	319 (34.3)	892 (29.9)	1.22 (1.04, 1.43)	0.012	2	0	1.61 (1.31, 2.00)	<0.0001
High Cholesterol	440 (49.8)	1487 (50.0)	0.99 (0.85, 1.15)	0.8921	48	8	0.79 (0.65, 0.96)	0.0183
Smoking:				0.8987	6	1		
Current Smoking	141 (15.2)	456 (15.3)	1.01 (0.81, 1.25)	0.9334			0.88 (0.67, 1.15)	0.3356
Former smoking	323 (34.9)	1015 (34.1)	1.04 (0.88, 1.22)	0.6469			0.95 (0.78, 1.16)	0.6202
Never Smoking	462 (49.9)	1508 (50.6)	Reference				Reference	

© 2021 Kittner SJ et al. *JAMA Network Open.*

**eTable 4. Lobar Intracerebral Hemorrhage: Univariable (Univ) and Multivariable (MV) Odds Ratios and P Values for All Variables in the Original Model Without Backwards Elimination (continued)**								
	Cases: N=932	Controls: N=2980	Univar OR (CI)	p-Value	Numbers Missing		MV OR (CI)	MV p-value
	No. (%)				Cases	Control		
Alcohol Use					13	22		
No/Rare use	594 (64.6)	1600 (54.1)	Reference				Reference	
Moderate Use	254 (27.6)	1234 (41.7)	0.55 (0.47, 0.65)	<0.0001			0.59 (0.48, 0.72)	<0.0001
Heavy Use	71 (7.7)	124 (4.2)	1.54 (1.14, 2.10)	0.0056			1.46 (1.01, 2.12)	0.0453
Cocaine/Amphetamine Use	24 (2.6)	27 (0.9)	2.89 (1.66, 5.03)	0.0002	0	0	3.82 (1.97, 7.38)	<0.0001
Antiplatelet use	57 (6.1)	112 (3.8)	1.67 (1.20, 2.32)	0.0022	0	0	1.41 (0.96, 2.09)	0.0829
Anticoagulant use	99 (10.6)	107 (3.6)	3.19 (2.40, 4.24)	<0.0001	0	0	2.99 (2.16, 4.14)	<0.0001
APOE: Presence of 2	165 (18.1)	442 (14.9)	1.26 (1.04, 1.54)	0.0209	22	22	1.36 (1.08, 1.72)	0.0088
APOE: Presence of 4	312 (34.3)	817 (27.6)	1.37 (1.17, 1.60)	0.0001	22	22	1.40 (1.16, 1.69)	0.0004
Lack of Medical Insurance	173 (18.6)	324 (10.9)	1.87 (1.53, 2.28)	<0.0001	0	0	2.42 (1.85, 3.16)	<0.0001

Abbreviations: OR, Odds Ratio; CI, Confidence Interval; SD, Standard Deviation; BMI, Body Mass Index; APOE, Apolipoprotein E.
[a] Twenty controls were not interviewed and are not included in this table. [b] Multivariable model included 812 cases and 2920 controls.

© 2021 Kittner SJ et al. *JAMA Network Open.*

eTable 5. Nonlobar Intracerebral Hemorrhage: Univariable (Univ) and Multivariable (MV) Odds Ratios and P Values for All Variables in the Original Model Without Backwards Elimination

	Cases: N=2066	Controls: N=2980[a]	Univar OR(CI)	p-Value	Numbers Missing		MV OR (CI)[b]	MV p-Value
					Cases	Control		
Age: Mean (SD)	60.1 (13.8)	61.6 (14.3)	0.99 (0.99, 1.00)	0.0002	0	0	1.00 (0.99, 1.00)	0.4323
Race:				0.0002	0	0		
Black	761 (36.8)	1000 (33.6)	1.31 (1.14, 1.51)	0.0001			0.82 (0.68, 0.99)	0.0389
Hispanic	726 (35.1)	980 (32.9)	1.28 (1.11, 1.47)	0.0006			0.81 (0.67, 0.98)	0.0274
White	579 (28.0)	1000 (33.6)	Reference				Reference	
Females	777 (37.6)	1233 (41.4)	0.85 (0.76, 0.96)	0.0072	0	0	0.91 (0.78, 1.06)	0.219
Ischemic History	204 (9.9)	40 (1.3)	8.05 (5.71, 11.36)	<0.0001	0	0	7.79 (5.34, 11.36)	<0.0001
Chronic Kidney Disease	187 (9.0)	123 (4.1)	2.31 (1.83, 2.92)	<0.0001	0	0	1.85 (1.39, 2.45)	<0.0001
Hypertension:				<0.0001	27	2		
No Hypertension	381 (18.7)	1389 (46.6)	Reference				Reference	
Treated Hypertension	911 (44.7)	1412 (47.4)	2.35 (2.04, 2.71)	<0.0001			2.92 (2.42, 3.52)	<0.0001
Untreated Hypertension	747 (36.6)	177 (5.9)	15.39 (12.61, 18.78)	<0.0001			13.52 (10.75, 17.00)	<0.0001
Diabetes	535 (26.0)	663 (22.3)	1.23 (1.08, 1.40)	0.0021	10	1	1.15 (0.96, 1.38)	0.1175
BMI:				<0.0001	51	7		
<18.5	45 (2.2)	25 (0.8)	2.29 (1.39, 3.78)	0.0012			1.91 (1.05, 3.48)	0.0331
18.5 to <25	532 (26.4)	677 (22.8)	Reference				Reference	
25 to <30	671 (33.3)	1068 (35.9)	0.80 (0.69, 0.93)	0.0033			0.74 (0.61, 0.89)	0.0013
>=30	767 (38.1)	1203 (40.5)	0.81 (0.70, 0.94)	0.0048			0.58 (0.47, 0.71)	<0.0001
Sleep Apnea Risk	860 (41.7)	892 (29.9)	1.67 (1.49, 1.88)	<0.0001	2	0	1.59 (1.34, 1.88)	<0.0001

© 2021 Kittner SJ et al. *JAMA Network Open.*

eTable 5. Nonlobar Intracerebral Hemorrhage: Univariable (Univ) and Multivariable (MV) Odds Ratios and P Values for All Variables in the Original Model Without Backwards Elimination (continued)

	Cases: N=2066	Controls: N=2980	Univar OR (CI)	p-Value	Numbers Missing		MV OR (CI)	MV p-Value
					Cases	Control		
High Cholesterol	782 (40.0)	1487 (50.0)	0.67 (0.59, 0.75)	<0.0001	113	8	0.58 (0.49, 0.68)	<0.0001
Smoking:				<0.0001	14	1		
Current Smoking	410 (20.0)	456 (15.3)	1.26 (1.08, 1.47)	0.0034			0.80 (0.65, 0.98)	0.0347
Former smoking	566 (27.6)	1015 (34.1)	0.78 (0.69, 0.89)	0.0002			0.76 (0.65, 0.90)	0.001
Never Smoking	1076 (52.4)	1508 (50.6)	Reference				Reference	
Alcohol Use:				<0.0001	53	22		
No/Rare use	1186 (58.9)	1600 (54.1)	Reference				Reference	
Moderate Use	606 (30.1)	1234 (41.7)	0.66 (0.59, 0.75)	<0.0001			0.69 (0.59, 0.80)	<0.0001
Heavy Use	221 (11.0)	124 (4.2)	2.40 (1.91, 3.03)	<0.0001			1.86 (1.39, 2.48)	<0.0001
Cocaine/Amphetamine Use	104 (5.0)	27 (0.9)	5.80 (3.78, 8.89)	<0.0001	0	0	4.21 (2.51, 7.05)	<0.0001
Antiplatelet use	79 (3.8)	112 (3.8)	1.02 (0.76, 1.37)	0.9045	0	0	1.18 (0.83, 1.68)	0.3615
Anticoagulant use	158 (7.6)	107 (3.6)	2.22 (1.73, 2.86)	<0.0001	0	0	3.14 (2.34, 4.22)	<0.0001
APOE: Presence of 2	276 (13.5)	442 (14.9)	0.89 (0.75, 1.04)	0.144	17	22	0.74 (0.60, 0.91)	0.0045
APOE: Presence of 4	583 (28.4)	817 (27.6)	1.04 (0.92, 1.18)	0.5178	17	22	0.96 (0.82, 1.12)	0.5827
Lack of Medical Insurance	658 (31.8)	324 (10.9)	3.83 (3.30, 4.44)	<0.0001	0	0	3.81 (3.12, 4.65)	<0.0001

Abbreviations: OR, Odds Ratio; CI, Confidence Interval; SD, Standard Deviation; BMI, Body Mass Index; APOE, Apolipoprotein E.
[a] Twenty controls were not interviewed and are not included in this table. [b] Multivariable model includes 1835 cases and 2920 controls.

© 2021 Kittner SJ et al. *JAMA Network Open.*

eTable 6. Lobar Intracerebral Hemorrhage, Black Subgroup, Univariable (Univ) and Multivariable (MV) Odds Ratios and P Values for All Variables in the Original Model Without Backwards Elimination

	Cases: N=238	Controls: N=1000	Univ OR (CI)	p-Value	Numbers Missing		MV OR (CI) [a]	MV p-Value
	No. (%)				Cases	Control		
Age: Mean (SD)	62.2 (15.2)	58.0 (12.3)	1.02 (1.01, 1.04)	<0.0001	0	0	1.01 (1.00, 1.03)	0.0812
Females	118 (49.6)	425 (42.5)	1.33 (1.00, 1.77)	0.0483	0	0	1.41 (0.98, 2.01)	0.0615
Ischemic History	23 (9.7)	14 (1.4)	7.53 (3.82, 14.88)	<0.0001	0	0	4.15 (1.73, 9.92)	0.0014
Chronic Kidney Disease	33 (13.9)	39 (3.9)	3.97 (2.44, 6.46)	<0.0001	0	0	2.63 (1.47, 4.72)	0.0012
Hypertension:					3	1		
No Hypertension	40 (17.0)	394 (39.4)	Reference				Reference	
Treated Hypertension	125 (53.2)	530 (53.0)	2.32 (1.59, 3.39)	<0.0001			2.12 (1.31, 3.43)	0.0022
Untreated Hypertension	70 (29.8)	75 (7.5)	9.19 (5.80, 14.57)	<0.0001			6.06 (3.51, 10.46)	<0.0001
Diabetes	72 (30.6)	223 (22.3)	1.54 (1.12, 2.11)	0.0074	3	1	1.28 (0.84, 1.94)	0.2607
BMI:					17	0		
<18.5	9 (4.1)	9 (0.9)	2.60 (0.99, 6.82)	0.0528			2.01 (0.67, 6.04)	0.2147
18.5 to <25	67 (30.3)	174 (17.4)	Reference				Reference	
25 to <30	62 (28.0)	343 (34.3)	0.47 (0.32, 0.69)	0.0001			0.52 (0.33, 0.81)	0.0045
>=30	83 (37.6)	474 (47.4)	0.46 (0.32, 0.66)	<0.0001			0.36 (0.22, 0.60)	<0.0001
Sleep Apnea Risk	86 (36.3)	333 (33.3)	1.14 (0.85, 1.53)	0.3825	1	0	1.54 (1.01, 2.35)	0.0443
High Cholesterol	88 (39.1)	435 (43.7)	0.83 (0.62, 1.11)	0.2119	13	4	0.54 (0.37, 0.81)	0.0026
Smoking:					1	0		
Current Smoking	59 (24.9)	252 (25.2)	0.97 (0.68, 1.38)	0.8739			0.76 (0.48, 1.19)	0.2267
Former smoking	66 (27.8)	283 (28.3)	0.97 (0.69, 1.36)	0.8517			0.83 (0.55, 1.26)	0.3896
Never Smoking	112 (47.3)	465 (46.5)	Reference				Reference	
Alcohol Use:					4	16		
No/Rare use	158 (67.5)	566 (57.5)	Reference				Reference	
Moderate Use	54 (23.1)	378 (38.4)	0.51 (0.37, 0.72)	<0.0001			0.64 (0.42, 0.95)	0.0278

© 2021 Kittner SJ et al. *JAMA Network Open.*

**eTable 6. Lobar Intracerebral Hemorrhage, Black Subgroup, Univariable (Univ) and Multivariable (MV) Odds Ratios and P Values for All Variables in the Original Model Without Backwards Elimination (continued)**								
	Cases: N=238	Controls: N=1000	Univ OR (CI)	p-Value	Numbers Missing		MV OR (CI)	MV p-Value
	No. (%)				Cases	Control		
Alcohol Use (continued)								
Heavy Use	22 (9.4)	40 (4.1)	1.97 (1.14, 3.41)	0.0155			1.86 (0.93, 3.69)	0.0775
Cocaine/Amphetamine Use	12 (5.0)	16 (1.6)	3.26 (1.52, 7.00)	0.0023	0	0	3.63 (1.40, 9.41)	0.0081
Antiplatelet use	13 (5.5)	24 (2.4)	2.35 (1.18, 4.69)	0.0153	0	0	1.52 (0.64, 3.59)	0.3400
Anticoagulant use	18 (7.6)	14 (1.4)	5.76 (2.82, 11.76)	<0.0001	0	0	6.84 (3.00, 15.58)	<0.0001
APOE: Presence of 2	47 (19.8)	193 (19.5)	1.02 (0.71, 1.46)	0.9174	1	12	1.01 (0.66, 1.55)	0.9579
APOE: Presence of 4	89 (37.6)	378 (38.3)	0.97 (0.72, 1.30)	0.841	1	12	0.91 (0.64, 1.29)	0.595
Lack of Medical Insurance	50 (21.0)	137 (13.7)	1.68 (1.17, 2.40)	0.0050	0	0	1.92 (1.20, 3.08)	0.0068

Abbreviations: OR, Odds Ratio; CI, Confidence Interval; SD, Standard Deviation; BMI, Body Mass Index; APOE, Apolipoprotein E.

[a] Multivariable model included 205 cases and 968 controls.

© 2021 Kittner SJ et al. *JAMA Network Open.*

eTable 7. Lobar Intracerebral Hemorrhage, Hispanic Subgroup, Univariable (Univ) and Multivariable (MV) Odds Ratios and P Values for All Variables in the Original Model Without Backwards Elimination

	Cases: N=274	Controls: N=980 [a]	Univ OR (CI)	p-Value	Numbers Missing		MV OR (CI) [b]	MV p-Value
					Cases	Controls		
Age: Mean (SD)	62.5 (15.7)	58.2 (14.6)	1.02 (1.01, 1.03)	<0.0001	0	0	1.02 (1.00, 1.03)	0.0097
Females	133 (48.5)	366 (37.4)	1.58 (1.21, 2.07)	0.0009	0	0	1.89 (1.34, 2.69)	0.0003
Ischemic History	22 (8.0)	15 (1.5)	5.62 (2.87, 10.98)	<0.0001	0	0	4.41 (1.97, 9.85)	0.0003
Chronic Kidney Disease	18 (6.6)	39 (4.0)	1.70 (0.95, 3.02)	0.0718	0	0	1.82 (0.93, 3.55)	0.0795
Hypertension:					9	1		
No Hypertension	78 (29.4)	514 (52.5)	Reference				Reference	
Treated Hypertension	121 (45.7)	402 (41.1)	1.98 (1.45, 2.71)	<0.0001			1.85 (1.20, 2.85)	0.0053
Untreated Hypertension	66 (24.9)	63 (6.4)	6.90 (4.54, 10.50)	<0.0001			6.20 (3.71, 10.34)	<0.0001
Diabetes	81 (29.9)	279 (28.5)	1.07 (0.80, 1.44)	0.6477	3	0	1.00 (0.67, 1.47)	0.9851
BMI:					7	3		
<18.5	5 (1.9)	2 (0.2)	5.32 (1.01, 27.94)	0.0485			8.29 (1.37, 50.08)	0.0212
18.5 to <25	87 (32.6)	185 (18.9)	Reference				Reference	
25 to <30	99 (37.1)	362 (37.0)	0.58 (0.42, 0.82)	0.0017			0.62 (0.41, 0.93)	0.0209
>=30	76 (28.5)	428 (43.8)	0.38 (0.26, 0.54)	<0.0001			0.31 (0.19, 0.51)	<0.0001
Sleep Apnea Risk	103 (37.7)	322 (32.9)	1.24 (0.94, 1.64)	0.133	1	0	1.44 (0.97, 2.14)	0.0738
High Cholesterol	120 (46.9)	479 (49.1)	0.92 (0.70, 1.21)	0.5303	18	4	0.83 (0.58, 1.19)	0.313
Smoking:					3	0		
Current Smoking	42 (15.5)	113 (11.5)	1.36 (0.91, 2.02)	0.1291			1.30 (0.79, 2.14)	0.2956
Former smoking	79 (29.2)	318 (32.4)	0.91 (0.67, 1.23)	0.5416			0.88 (0.60, 1.28)	0.5007
Never Smoking	150 (55.4)	549 (56.0)	Reference				Reference	
Alcohol Use:					6	3		
No/Rare use	181 (67.5)	592 (60.6)	Reference				Reference	
Moderate Use	60 (22.4)	355 (36.3)	0.55 (0.40, 0.76)	0.0003			0.75 (0.51, 1.11)	0.1515
Heavy Use	27 (10.1)	30 (3.1)	2.94 (1.70, 5.08)	0.0001			3.06 (1.49, 6.31)	0.0024

© 2021 Kittner SJ et al. *JAMA Network Open.*

eTable 7. Lobar Intracerebral Hemorrhage, Hispanic Subgroup, Univariable (Univ) and Multivariable (MV) Odds Ratios and P Values for All Variables in the Original Model Without Backwards Elimination (continued)

	Cases: N=274	Controls: N=980	Univ OR (CI)	p-Value	Numbers Missing		MV OR (CI)	MV p-Value
					Cases	Controls		
Cocaine/Amphetamine Use	10 (3.6)	9 (0.9)	4.09 (1.64, 10.16)	0.0024	0	0	6.22 (2.17, 17.80)	0.0007
Antiplatelet use	13 (4.7)	36 (3.7)	1.31 (0.68, 2.50)	0.4197	0	0	0.98 (0.44, 2.22)	0.967
Anticoagulant use	26 (9.5)	26 (2.6)	3.85 (2.20, 6.74)	<0.0001	0	0	4.50 (2.35, 8.60)	<0.0001
APOE: Presence of 2	24 (8.9)	76 (7.8)	1.15 (0.71, 1.86)	0.5647	4	7	1.00 (0.55, 1.80)	0.9882
APOE: Presence of 4	72 (26.7)	199 (20.4)	1.41 (1.04, 1.93)	0.0291	4	7	1.21 (0.82, 1.78)	0.3316
Lack of Medical Insurance	85 (31.0)	146 (14.9)	2.57 (1.88, 3.50)	<0.0001	0	0	3.26 (2.13, 4.97)	<0.0001

Abbreviations: OR, Odds Ratio; CI, Confidence Interval; SD, Standard Deviation; BMI, Body Mass Index; APOE, Apolipoprotein E.

[a] Twenty controls were not interviewed and are not included in this table. [b] Multivariable model included 236 cases and 962 controls.

© 2021 Kittner SJ et al. *JAMA Network Open.*

eTable 8. Lobar Intracerebral Hemorrhage, White Subgroup, Univariable (Univ) and Multivariable (MV) Odds Ratios and P Values for All Variables in the Original Model Without Backwards Elimination

	Cases: N=420	Controls: N=1000	Univar OR (CI)	p-Value	Numbers Missing		MV OR (CI)[a]	MV p-Value
					Cases	Controls		
Age: Mean (SD)	71.0 (13.3)	68.6 (13.2)	1.01 (1.00, 1.02)	0.0022	0	0	1.00 (0.99, 1.01)	0.8052
Females	206 (49.0)	442 (44.2)	1.22 (0.97, 1.53)	0.0944	0	0	1.18 (0.90, 1.56)	0.2352
Ischemic History	36 (8.6)	11 (1.1)	8.43 (4.25, 16.73)	<0.0001	0	0	6.85 (3.26, 14.38)	<0.0001
Chronic Kidney Disease	21 (5.0)	45 (4.5)	1.12 (0.66, 1.90)	0.6831	0	0	0.95 (0.51, 1.77)	0.8826
Hypertension:					3	0		
No Hypertension	139 (33.3)	481 (48.1)	Reference				Reference	
Treated Hypertension	202 (48.4)	480 (48.0)	1.46 (1.13, 1.87)	0.0032			1.41 (1.02, 1.94)	0.0365
Untreated Hypertension	76 (18.2)	39 (3.9)	6.74 (4.39, 10.36)	<0.0001			5.05 (3.09, 8.25)	<0.0001
Diabetes	70 (16.7)	161 (16.1)	1.05 (0.77, 1.43)	0.7623	2	0	0.99 (0.68, 1.44)	0.9707
BMI:					15	4		
<18.5	9 (2.2)	14 (1.4)	1.26 (0.54, 2.98)	0.5951			0.82 (0.29, 2.32)	0.7043
18.5 to <25	162 (40.0)	318 (31.9)	Reference				Reference	
25 to <30	139 (34.3)	363 (36.4)	0.75 (0.57, 0.99)	0.0397			0.62 (0.45, 0.85)	0.0034
>=30	95 (23.5)	301 (30.2)	0.62 (0.46, 0.84)	0.0017			0.43 (0.29, 0.64)	<0.0001
Sleep Apnea Risk	130 (31.0)	237 (23.7)	1.44 (1.12, 1.86)	0.0045	0	0	1.76 (1.26, 2.45)	0.0008
High Cholesterol	232 (57.6)	573 (57.3)	1.01 (0.80, 1.28)	0.9268	17	0	0.93 (0.69, 1.26)	0.6436
Smoking:					2	1		
Current Smoking	40 (9.6)	91 (9.1)	1.09 (0.72, 1.63)	0.6917			0.77 (0.46, 1.29)	0.3169
Former smoking	178 (42.6)	414 (41.4)	1.06 (0.84, 1.35)	0.6241			1.10 (0.83, 1.45)	0.5208
Never Smoking	200 (47.8)	494 (49.4)	Reference				Reference	
Alcohol Use:					3	3		
No/Rare use	255 (61.1)	442 (44.3)	Reference				Reference	
Moderate Use	140 (33.6)	501 (50.2)	0.48 (0.38, 0.62)	<0.0001			0.50 (0.38, 0.67)	<0.0001
Heavy Use	22 (5.3)	54 (5.4)	0.71 (0.42, 1.19)	0.189			0.87 (0.47, 1.59)	0.6411

© 2021 Kittner SJ et al. *JAMA Network Open.*

eTable 8. Lobar Intracerebral Hemorrhage, White Subgroup, Univariable (Univ) and Multivariable (MV) Odds Ratios and P Values for All Variables in the Original Model Without Backwards Elimination (continued)								
	Cases: N=420	Controls: N=1000	Univar OR (CI)	p-Value	Numbers Missing		MV OR (CI)	MV p-Value
					Cases	Controls		
Cocaine/Amphetamine Use	2 (0.5)	2 (0.2)	2.39 (0.34, 17.00)	0.3851	0	0	0.68 (0.05, 8.86)	0.7705
Antiplatelet use	31 (7.4)	52 (5.2)	1.45 (0.92, 2.30)	0.1115	0	0	1.42 (0.82, 2.47)	0.211
Anticoagulant use	55 (13.1)	67 (6.7)	2.10 (1.44, 3.06)	0.0001	0	0	2.24 (1.45, 3.47)	0.0003
APOE: Presence of 2	94 (23.3)	173 (17.4)	1.45 (1.09, 1.92)	0.0102	17	3	1.80 (1.30, 2.50)	0.0004
APOE: Presence of 4	151 (37.5)	240 (24.1)	1.89 (1.47, 2.42)	<0.0001	17	3	1.85 (1.39, 2.46)	<0.0001
Lack of Medical Insurance	38 (9.0)	41 (4.1)	2.33 (1.47, 3.68)	0.0003	0	0	2.68 (1.48, 4.82)	0.0011

Abbreviations: OR, Odds Ratio; CI, Confidence Interval; SD, Standard Deviation; BMI, Body Mass Index; APOE, Apolipoprotein E.

[a] Multivariable model included 371 cases and 990 controls.

© 2021 Kittner SJ et al. *JAMA Network Open.*

eTable 9. Nonlobar Intracerebral Hemorrhage, Black Subgroup, Univariable (Univ) and Multivariable (MV) Odds Ratios and P Values for All Variables in the Original Model Without Backwards Elimination

	Cases: N=761 [a]	Controls: N=1000	Univ OR (CI)	p-Value	Numbers Missing		MV OR (CI) [b]	MV p-Value
					Cases	Control		
Age: Mean (SD)	56.7 (11.6)	58.0 (12.3)	0.99 (0.98, 1.00)	0.0272	0	0	0.99 (0.98, 1.00)	0.1693
Females	306 (40.2)	425 (42.5)	0.91 (0.75, 1.10)	0.3347	0	0	0.93 (0.72, 1.20)	0.5911
Ischemic History	76 (10.0)	14 (1.4)	7.81 (4.38, 13.93)	<0.0001	0	0	8.53 (4.43, 16.43)	<0.0001
Chronic Kidney Disease	74 (9.7)	39 (3.9)	2.65 (1.78, 3.96)	<0.0001	0	0	1.97 (1.22, 3.19)	0.0059
Hypertension:					10	1		
No Hypertension	95 (12.6)	394 (39.4)	Reference				Reference	
Treated Hypertension	360 (47.9)	530 (53.0)	2.82 (2.17, 3.66)	<0.0001			3.85 (2.72, 5.44)	<0.0001
Untreated Hypertension	296 (39.4)	75 (7.5)	16.37 (11.67, 22.96)	<0.0001			15.30 (10.36, 22.60)	<0.0001
Diabetes	168 (22.2)	223 (22.3)	0.99 (0.79, 1.25)	0.9602	5	1	1.02 (0.75, 1.39)	0.8979
BMI:					21	0		
<18.5	15 (12.0)	9 (0.9)	1.49 (0.64, 3.48)	0.3608			0.94 (0.32, 2.79)	0.9082
18.5 to <25	195 (26.4)	174 (17.4)	Reference				Reference	
25 to <30	220 (29.7)	343 (34.3)	0.57 (0.44, 0.75)	<0.0001			0.67 (0.48, 0.94)	0.0224
>=30	310 (41.9)	474 (47.4)	0.58 (0.46, 0.75)	<0.0001			0.50 (0.35, 0.72)	0.0002
Sleep Apnea Risk	329 (43.3)	333 (33.3)	1.53 (1.26, 1.86)	<0.0001	1	0	1.75 (1.32, 2.31)	0.0001
High Cholesterol	243 (33.7)	435 (43.7)	0.66 (0.54, 0.80)	<0.0001	40	4	0.62 (0.47, 0.82)	0.0007
Smoking:					8	0		
Current Smoking	218 (29.0)	252 (25.2)	1.07 (0.85, 1.34)	0.5747			0.65 (0.48, 0.89)	0.0068
Former smoking	158 (21.0)	283 (28.3)	0.69 (0.54, 0.87)	0.0021			0.62 (0.46, 0.84)	0.0022
Never Smoking	377 (50.1)	465 (46.5)	Reference					
Alcohol Use:					27	16		
No/Rare use	426 (58.0)	566 (57.5)	Reference				Reference	
Moderate Use	220 (30.0)	378 (38.4)	0.77 (0.63, 0.95)	0.0156			0.81 (0.62, 1.06)	0.1269
Heavy Use	88 (12.0)	40 (4.1)	2.92 (1.97, 4.34)	<0.0001			2.19 (1.32, 3.62)	0.0023

© 2021 Kittner SJ et al. *JAMA Network Open.*

eTable 9. Nonlobar Intracerebral Hemorrhage, Black Subgroup, Univariable (Univ) and Multivariable (MV) Odds Ratios and P Values for All Variables in the Original Model Without Backwards Elimination(continued)

	Cases: N=761	Controls: N=1000	Univ OR (CI)	p-Value	Numbers Missing		MV OR (CI)	MV p-Value
					Cases	Control		
Cocaine/Amphetamine Use	60 (7.9)	16 (1.6)	5.26 (3.01, 9.21)	<0.0001	0	0	4.96 (2.47, 9.98)	<0.0001
Antiplatelet use	19 (2.5)	24 (2.4)	1.04 (0.57, 1.92)	0.8961	0	0	0.92 (0.43, 1.97)	0.8307
Anticoagulant use	31 (4.1)	14 (1.4)	2.99 (1.58, 5.66)	0.0008	0	0	5.54 (2.61, 11.75)	<0.0001
APOE: Presence of 2	150 (19.8)	193 (19.5)	1.02 (0.80, 1.29)	0.873	5	12	0.87 (0.64, 1.18)	0.3644
APOE: Presence of 4	301 (39.8)	378 (38.3)	1.07 (0.88, 1.30)	0.5087	5	12	0.97 (0.75, 1.24)	0.7884
Lack of Insurance	282 (37.1)	137 (13.7)	3.71 (2.94, 4.68)	<0.0001	0	0	3.44 (2.53, 4.67)	<0.0001

Abbreviations: OR, Odds Ratio; CI, Confidence Interval; SD, Standard Deviation; BMI, Body Mass Index; APOE, Apolipoprotein E.

[a] One case was not interviewed and is not included in this table. [b] Multivariable model included 672 cases and 968 controls.

© 2021 Kittner SJ et al. *JAMA Network Open.*

eTable 10. Nonlobar Intracerebral Hemorrhage, Hispanic Subgroup, Univariable (Univ) and Multivariable (MV) Odds Ratios and P Values for All Variables in the Original Model Without Backwards Elimination

	Cases: N=726	Controls: N=980 [a]	Univ OR (CI)	p-Value	Numbers Missing		MV OR (CI) [b]	MV p-Value
					Cases	Control		
Age: Mean (SD)	57.6 (13.4)	58.2 (14.6)	1.00 (0.99, 1.00)	0.3887	0	0	1.01 (1.00, 1.02)	0.2476
Females	240 (33.1)	366 (37.4)	0.83 (0.68, 1.01)	0.0674	0	0	1.14 (0.87, 1.51)	0.3379
Ischemic History	77 (10.6)	15 (1.5)	7.63 (4.35, 13.39)	<0.0001	0	0	7.18 (3.85, 13.40)	<0.0001
Chronic Kidney Disease	77 (10.6)	39 (4.0)	2.86 (1.92, 4.26)	<0.0001	0	0	2.65 (1.62, 4.33)	<0.0001
Hypertension:					9	1		
No Hypertension	147 (20.5)	514 (52.5)	Reference				Reference	
Treated Hypertension	273 (38.1)	402 (41.1)	2.38 (1.87, 3.02)	<0.0001			3.72 (2.62, 5.28)	<0.0001
Untreated Hypertension	297 (41.4)	63 (6.4)	16.48 (11.88, 22.88)	<0.0001			16.82 (11.29, 25.05)	<0.0001
Diabetes	227 (31.4)	279 (28.5)	1.15 (0.93, 1.42)	0.1915	3	0	1.06(0.78, 1.42)	0.7235
BMI:					12	3		
<18.5	15 (2.1)	2 (0.2)	9.76 (2.20, 43.33)	0.0027			5.88 (1.18, 29.22)	0.0304
18.5 to <25	142 (19.9)	185 (18.9)	Reference				Reference	
25 to <30	286 (40.1)	362 (37.0)	1.03 (0.79, 1.35)	0.8328			0.93 (0.66, 1.31)	0.6681
>=30	271 (38.0)	428 (43.8)	0.82 (0.63, 1.08)	0.1567			0.68 (0.47, 1.00)	0.0497
Sleep Apnea Risk	305 (42.0)	322 (32.9)	1.48 (1.21, 1.81)	0.0001	0	0	1.22 (0.90, 1.65)	0.207
High Cholesterol	257 (37.8)	479 (49.1)	0.63 (0.52, 0.77)	<0.0001	47	4	0.53 (0.40, 0.70)	<0.0001
Smoking:					3	0		
Current Smoking	101 (14.0)	113 (11.5)	1.20 (0.89, 1.62)	0.2300			0.78 (0.52, 1.16)	0.2208
Former smoking	213 (29.5)	318 (32.4)	0.90 (0.72, 1.12)	0.3336			0.92 (0.69, 1.22)	0.538
Never Smoking	409 (56.6)	549 (56.0)	Reference				Reference	
Alcohol Use:					19	3		
No/Rare use	416 (58.8)	592 (60.6)	Reference				Reference	
Moderate Use	204 (28.8)	355 (36.3)	0.82 (0.66, 1.01)	<0.0642			0.98 (0.74, 1.31)	0.9083
Heavy Use	87 (12.3)	30 (3.1)	4.13 (2.68, 6.37)	<0.0001			4.62 (2.68, 7.99)	<0.0001

© 2021 Kittner SJ et al. *JAMA Network Open.*

eTable 10. Nonlobar Intracerebral Hemorrhage, Hispanic Subgroup, Univariable (Univ) and Multivariable (MV) Odds Ratios and P Values for All Variables in the Original Model Without Backwards Elimination (continued)								
	Cases: N=726	Controls: N=980	Univ OR (CI)	p-Value	Numbers Missing		MV OR (CI)	MV p-Value
					Cases	Control		
Cocaine/Amphetamine Use	28 (3.9)	9 (0.9)	4.33 (2.03, 9.23)	0.0001	0	0	3.26 (1.26, 8.44)	0.0147
Antiplatelet use	24 (3.3)	36 (3.7)	0.90 (0.53, 1.52)	0.6837	0	0	1.01 (0.53, 1.94)	0.9662
Anticoagulant use	40 (5.5)	26 (2.6)	2.14 (1.29, 3.54)	0.0031	0	0	2.68 (1.48, 4.86)	0.0012
APOE: Presence of 2	48 (6.6)	76 (7.8)	0.84 (0.58, 1.22)	0.3516	1	7	0.64 (0.39, 1.04)	0.074
APOE: Presence of 4	143 (19.7)	199 (20.4)	0.96 (0.75, 1.22)	0.7119	1	7	0.85 (0.62, 1.17)	0.3215
Lack of Insurance	302 (41.6)	146 (14.9)	4.07 (3.23, 5.12)	<0.0001	0	0	5.28 (3.81, 7.30)	<0.0001

Abbreviations: OR, Odds Ratio; CI, Confidence Interval; SD, Standard Deviation; BMI, Body Mass Index; APOE, Apolipoprotein E.
[a] Twenty controls were not interviewed and are not included in this table. [b] Multivariable model included 648 cases and 962 controls.

© 2021 Kittner SJ et al. *JAMA Network Open.*

eTable 11. Nonlobar Intracerebral Hemorrhage, White Subgroup, Univariable (Univ) and Multivariable (MV) Odds Ratios and P Values for All Variables in the Original Model Without Backwards Elimination

	Cases: N=579 [a]	Controls: N=1000	Univ OR (CI)	p-Value	Numbers Missing		MV OR (CI) [b]	MV p-Value
					Cases	Control		
Age: Mean (SD)	67.8 (14.1)	68.6 (13.2)	1.00 (0.99, 1.00)	0.2491	0	0	1.00 (0.99, 1.01)	0.7208
Females	231 (39.9)	442 (44.2)	0.84 (0.68, 1.03)	0.0958	0	0	0.76 (0.58, 0.99)	0.039
Ischemic History	51 (8.8)	11 (1.1)	8.68 (4.49, 16.80)	<0.0001	0	0	7.61 (3.72, 15.54)	<0.0001
Chronic Kidney Disease	36 (6.2)	45 (4.5)	1.41 (0.90, 2.21)	0.1376	0	0	1.19 (0.70, 2.00)	0.5175
Hypertension:					8	0		
No Hypertension	139 (24.3)	481 (48.1)	Reference				Reference	
Treated Hypertension	278 (48.7)	480 (48.0)	2.00 (1.58, 2.55)	<0.0001			2.07 (1.52, 2.82)	<0.0001
Untreated Hypertension	154 (27.0)	39 (3.9)	13.66 (9.17, 20.36)	<0.0001			11.84 (7.55, 18.57)	<0.0001
Diabetes	140 (24.3)	161 (16.1)	1.67 (1.30, 2.15)	<0.0001	2	0	1.40 (1.00, 1.94)	0.0476
BMI:					18	4		
<18.5	15 (2.7)	14 (1.4)	1.75 (0.82, 3.70)	0.1447			1.67 (0.71, 3.94)	0.2437
18.5 to <25	195 (34.8)	318 (31.9)	Reference				Reference	
25 to <30	165 (29.4)	363 (36.4)	0.74 (0.57, 0.96)	0.0220			0.65 (0.48, 0.89)	0.0069
>=30	186 (33.2)	301 (30.2)	1.01 (0.78, 1.30)	0.9529			0.58 (0.41, 0.84)	0.0034
Sleep Apnea Risk	226 (39.1)	237 (23.7)	2.07 (1.66, 2.58)	<0.0001	1	0	1.80 (1.33, 2.43)	0.0002
High Cholesterol	282 (51.0)	573 (57.3)	0.78 (0.63, 0.96)	0.0169	26	0	0.55 (0.42, 0.73)	<0.0001
Smoking:					3	1		
Current Smoking	91 (15.8)	91 (9.1)	1.70 (1.23, 2.34)	0.0013			1.16 (0.76, 1.77)	0.4835
Former smoking	195 (33.8)	414 (41.4)	0.80 (0.64, 1.00)	0.0536			0.80 (0.61, 1.04)	0.1006
Never Smoking	290 (50.4)	494 (49.4)	Reference				Reference	
Alcohol Use:					7	3		
No/Rare use	344 (60.1)	442 (44.3)	Reference				Reference	
Moderate Use	182 (31.8)	501 (50.2)	0.47 (0.37, 0.58)	<0.0001			0.47 (0.36, 0.62)	<0.0001
Heavy Use	46 (8.0)	54 (5.4)	1.10 (0.72, 1.66)	0.6717			0.81 (0.48, 1.38)	0.4389

© 2021 Kittner SJ et al. *JAMA Network Open.*

eTable 11. Nonlobar Intracerebral Hemorrhage, White Subgroup, Univariable (Univ) and Multivariable (MV) Odds Ratios and P Values for All Variables in the Original Model Without Backwards Elimination (continued)								
	Cases: N=579	Controls: N=1000	Univ OR (CI)	p-Value	Numbers Missing		MV OR (CI)	MV p-Value
					Cases	Control		
Cocaine/Amphetamine Use	16 (2.8)	2 (0.2)	14.17 (3.25, 61.82)	0.0004	0	0	6.46 (1.29, 32.31)	0.0232
Antiplatelet use	36 (6.2)	52 (5.2)	1.21 (0.78, 1.87)	0.3962	0	0	1.37 (0.81, 2.30)	0.2365
Anticoagulant use	87 (15.0)	67 (6.7)	2.46 (1.76, 3.45)	<0.0001	0	0	3.02 (2.04, 4.48)	<0.0001
APOE: Presence of 2	78 (13.7)	173 (17.4)	0.76 (0.57, 1.01)	0.0612	11	3	0.65 (0.46, 0.93)	0.0175
APOE: Presence of 4	139 (24.5)	240 (24.1)	1.02 (0.80, 1.30)	0.8589	11	3	0.99 (0.74, 1.33)	0.9631
Lack of Insurance	74 (12.8)	41 (4.1)	3.43 (2.30, 5.09)	<0.0001	0	0	2.35 (1.38, 3.99)	0.0015

Abbreviations: OR, Odds Ratio; CI, Confidence Interval; SD, Standard Deviation; BMI, Body Mass Index; APOE, Apolipoprotein E.

[a] One case was not interviewed and is not included in this table. [b] Multivariable model included 515 cases and 990 controls.

© 2021 Kittner SJ et al. *JAMA Network Open.*
